# Investigation of Unsafe Construction Site Conditions Using Deep Learning Algorithms Using Unmanned Aerial Vehicles

**DOI:** 10.3390/s24206737

**Published:** 2024-10-14

**Authors:** Sourav Kumar, Mukilan Poyyamozhi, Balasubramanian Murugesan, Narayanamoorthi Rajamanickam, Roobaea Alroobaea, Waleed Nureldeen

**Affiliations:** 1 Department of Civil Engineering, SRM Institute of Science and Technology, Kattankulathur, Chennai 603203, India; sourav.kumar390@gmail.com (S.K.); mp6481@srmist.edu.in (M.P.); 2 Department of Electrical and Electronics Engineering, SRM Institute of Science and Technology, Kattankulathur, Chennai 603203, India; narayanr@srmist.edu.in; 3 Department of Computer Science, College of Computers and Information Technology, Taif University, P.O. Box 11099, Taif 21944, Saudi Arabia; r.robai@tu.edu.sa; 4 General Subject Department, University of Business and Technology, Jeddah 23435, Saudi Arabia

**Keywords:** Unmanned Aerial Vehicle, unsafe site conditions, object detection, tensor flow, automatic detection, image recognition

## Abstract

The rapid adoption of Unmanned Aerial Vehicles (UAVs) in the construction industry has revolutionized safety, surveying, quality monitoring, and maintenance assessment. UAVs are increasingly used to prevent accidents caused by falls from heights or being struck by falling objects by ensuring workers comply with safety protocols. This study focuses on leveraging UAV technology to enhance labor safety by monitoring the use of personal protective equipment, particularly helmets, among construction workers. The developed UAV system utilizes the tensorflow technique and an alert system to detect and identify workers not wearing helmets. Employing the high-precision, high-speed, and widely applicable Faster R-CNN method, the UAV can accurately detect construction workers with and without helmets in real-time across various site conditions. This proactive approach ensures immediate feedback and intervention, significantly reducing the risk of injuries and fatalities. Additionally, the implementation of UAVs minimizes the workload of site supervisors by automating safety inspections and monitoring, allowing for more efficient and continuous oversight. The experimental results indicate that the UAV system’s high precision, recall, and processing capabilities make it a reliable and cost-effective solution for improving construction site safety. The precision, mAP, and FPS of the developed system with the R-CNN are 93.1%, 58.45%, and 27 FPS. This study demonstrates the potential of UAV technology to enhance safety compliance, protect workers, and improve the overall quality of safety management in the construction industry.

## 1. Introduction

The construction sector is a crucial catalyst for both national and international economic expansion, considerably impacting infrastructure development, urbanization, and the broader economy. Nonetheless, the industry continues to be one of the most dangerous work environments, where even little errors may result in significant injuries or deaths. Notwithstanding ongoing developments in safety technology, such as Personal Protective Equipment (PPE) and more rigorous regulations, the sector continues to grapple with the complete eradication of accidents. To mitigate these ongoing safety problems, innovative technology such as UAVs is being incorporated into construction safety processes [1]. Nonetheless, the use of UAV technology presents a distinct array of issues that need thorough examination. This research seeks to examine the obstacles associated with the application and potential of UAVs in enhancing safety on building sites, while also resolving the inherent difficulties. Construction sites are intrinsically hazardous, including several risk factors such as falls from elevations, scaffolding collapses, inappropriate heavy machinery operation, and neglect of appropriate safety equipment. Notwithstanding extensive attempts to enhance worker safety, data reveal that construction remains responsible for a considerable proportion of occupational injuries and deaths globally [2]. According to the U.S. Bureau of Labor Statistics (BLS), 84% of employees who had impact injuries were not using appropriate PPE [3]. These numbers underscore a fundamental problem in the construction industry: worker non-adherence to safety regulations, often influenced by negligence, discomfort, or time constraints. In this environment, conventional techniques of monitoring safety compliance, such as site supervisors doing physical inspections, have shown inadequacy in both effectiveness and efficiency [4]. Manual inspections are labor-intensive and sometimes inadequate in identifying all possible hazards, especially in extensive projects or intricate locations where risks may develop quickly. A novel approach to construction safety is essential, using contemporary technology to provide real-time data, improved supervision, and rapid reaction capabilities. UAVs have surfaced as a viable answer to these challenges; nevertheless, their integration into the industry adds more complications [5]. UAVs, generally known as drones, have several advantages for construction safety, such as real-time aerial monitoring, automated assessments, and data acquisition. Fitted with high-resolution cameras and sensors, UAVs can detect risks from locations that are hard or dangerous for human workers to reach, like scaffolding, building peripheries, and crane summits. They may provide a thorough and extensive overview of the site, enabling safety managers to swiftly identify non-compliance with PPE regulations, equipment failures, and other safety hazards. In the occurrence of an accident, UAVs may provide essential situational awareness by assessing the accident site and directing first responders to affected individuals. The ability for immediate data gathering and real-time monitoring has considerable promise for enhancing worker safety. Nonetheless, the effective use of UAV technology in construction environments presents certain problems. The primary concern is adherence to regulations. Numerous nations impose stringent aviation laws on the use of UAVs, especially in densely populated or metropolitan regions, where construction activities are often concentrated. These rules sometimes set limitations on the locations, timings, and methods of drone operation, potentially conflicting with the requirements of a building site that needs ongoing surveillance [6]. Compliance with these standards often requires a specialized license, potentially elevating business expenses and introducing logistical challenges [7]. Furthermore, the use of UAVs necessitates the presence of highly skilled professionals for their operation and maintenance. Despite the user-friendly design of many contemporary UAVs, proficient operation requires a specific skill set, especially when managing bigger, more intricate drones equipped with sophisticated functionalities like thermal imaging, 3D mapping, and autonomous flying capabilities [8]. This prompts inquiries over the preparedness of the construction sector, which traditionally relies on manual labor, to include highly specialized positions. The problem lies in ensuring that UAV operators possess not just flying proficiency but also training in construction safety regulations to accurately evaluate and respond to the gathered data. Although UAVs have significant potential for enhancing construction site safety, they possess inherent technological limitations. The battery life is a considerable difficulty. Most commercial drones possess a restricted flying duration, generally ranging from 20 to 40 min—before requiring recharging. For a construction site that functions for prolonged durations, often from early morning to late afternoon, this constraint necessitates either regular recharging or several UAVs, both of which may substantially elevate costs and logistical intricacies [9]. A further technological difficulty is the incorporation of UAV data into current safety management systems. UAVs may provide extensive data, including high-definition video, thermal imagery, and sensor measurements. Collecting this data are only the first step; it must then be processed, evaluated, and acted upon in real time [10]. Numerous construction firms lack the necessary infrastructure to efficiently handle and use this data. Establishing the requisite data processing systems may be both labor-intensive and costly, especially for smaller enterprises or projects with constrained budgets. Moreover, UAVs are vulnerable to environmental factors, especially meteorological conditions [11]. Precipitation, high winds, and severe temperatures may adversely affect the operational safety and efficacy of drones. In building sites situated in areas with erratic weather conditions, these constraints might impede the regular utilization of UAVs, necessitating firms to establish contingency plans for instances when drones are inoperable. Moreover, UAVs are restricted to a certain operational range from the operator or base station, necessitating the deployment of many drones or base stations on extensive building sites to provide comprehensive coverage [12]. A significant albeit intangible hurdle to the adoption of UAVs in construction is the industry’s aversion to automation. Construction has historically been a labor-intensive sector, and several workers and supervisors may be reluctant to adopt technology that may be seen as jeopardizing job security or introducing unwarranted complexity to existing processes [13]. A widespread lack of information or comprehension of the advantages of UAVs may result in opposition among both management and employees. To surmount this opposition, firms must prioritize educating their staff on the benefits of UAV technology, including both safety and enhancements in efficiency and production. UAVs can discover labor allocation bottlenecks and provide data to improve operations, enhancing project deadlines and minimizing costs. Highlighting the beneficial effects of UAVs on project performance might alleviate apprehensions over job displacement and promote a more receptive attitude towards the use of new technology [14]. The extensive use of UAVs also prompts significant regulatory and ethical problems. Privacy is a significant issue, especially on building sites next to residential zones. UAVs outfitted with high-resolution cameras may unintentionally record images of adjacent private properties or persons, hence presenting concerns around data protection and privacy infringements [15]. Construction firms must guarantee that their use of UAVs adheres to all relevant privacy regulations, and they may need to formulate explicit protocols for drone operation to prevent legal issues. The rising utilization of UAVs, with privacy issues, underscores the need for stringent cybersecurity protocols. UAVs are fundamentally airborne computing systems, and like any digital apparatus, they are vulnerable to hacking or illegal intrusion [16]. Compromise of a drone’s control systems or data streams might result in grave repercussions, including the theft of key project information or the intentional collision of a UAV with personnel or equipment. Prioritizing the security of UAV operations is essential for any organization intending to include drones in their construction safety practices [17]. Despite the many hurdles linked to the deployment of UAVs in the construction sector, their prospective advantages render them an invaluable asset for improving worker safety and site management. As technology advances, several existing constraints, such as battery longevity, data processing efficiency, and regulatory obstacles, are expected to be alleviated. The development of more efficient battery systems and autonomous flying capabilities may enable UAVs to function for extended durations with less human oversight, hence enhancing their use for ongoing site monitoring [18]. Furthermore, the incorporation of artificial intelligence (AI) into UAV systems has the potential to transform their efficacy in danger identification and adherence to safety regulations [19,20]. AI-driven drones may assess site conditions in real time, automatically identify safety infractions, and even forecast probable mishaps based on trends in worker conduct or equipment functionality. This proactive strategy for safety management may prevent incidents before their occurrence rather than only addressing them post-event [21]. The integration of UAVs corresponds with overarching trends in the construction sector towards automation and the use of digital technology. As the industry advances towards further automation via robotics, Building Information Modeling (BIM), and the Internet of Things (IOT), UAVs are expected to become a fundamental component of the construction ecosystem [22]. Their capacity to provide real-time data and insights will enhance other digital technologies, contributing to the development of safer, more efficient, and more sustainable building projects. The use of UAVs in construction site safety management is a viable answer to several critical difficulties within the sector, especially in augmenting worker safety, strengthening site surveillance, and maximizing project efficiency. Nonetheless, the implementation of UAVs presents several challenges. Regulatory compliance, technological constraints, industrial opposition, and ethical dilemmas provide substantial obstacles that must be resolved to guarantee the effective deployment of this technology [23]. Notwithstanding these limitations, the prospective advantages of UAVs in the construction industry render them an invaluable asset for future safety management, and ongoing technological developments are expected to mitigate several existing impediments. As UAVs gain prominence, they possess the capacity to revolutionize labor safety management, fostering a safer, more efficient, and ultimately more sustainable construction sector [24]. This research encounters several substantial problems, especially focused on the incorporation of UAVs into construction site safety management. A primary challenge is maneuvering through intricate legal frameworks that impose limitations on UAV operations in urban and high-risk zones. Furthermore, the need for proficient individuals to operate UAVs and analyze their data poses a training and resource problem, particularly for construction firms that have historically depended on manual labor. Technical constraints, like limited battery longevity, susceptibility to weather conditions, and data processing demands, exacerbate the challenges of implementation. Furthermore, the industry’s opposition to automation, stemming from concerns about job loss and a lack of expertise with new technology, constitutes an additional obstacle. Ethical considerations, including privacy and cybersecurity threats, must be addressed to guarantee appropriate UAV utilization. Despite these hurdles, UAVs provide transformational potential for enhancing construction site safety; yet, their effective integration necessitates addressing these complex issues.

The major contributions in this paper are as follows:

- The study presents a UAV-based system that uses the Faster R-CNN method to accurately detect construction workers’ use of PPE, particularly helmets, in real-time and gives an alert to the site supervisor for ensuring immediate feedback and intervention to prevent accidents.
- The developed system is integrated with the low-cost UAV, and the real-time tests are performed to validate and achieve high precision and recall in detecting workers without helmets.
- The user-friendly API using the Sinch application was developed to give indication and alter to the site supervisor and helps to reduce the risk of injuries and fatalities.
- Overall, the RCCN integrated UAV system with the Sinch app reduces the workload on site supervisors, enabling more efficient and continuous oversight, which enhances the overall safety management on construction sites.

## 2. Methodology

**Figure 1:**
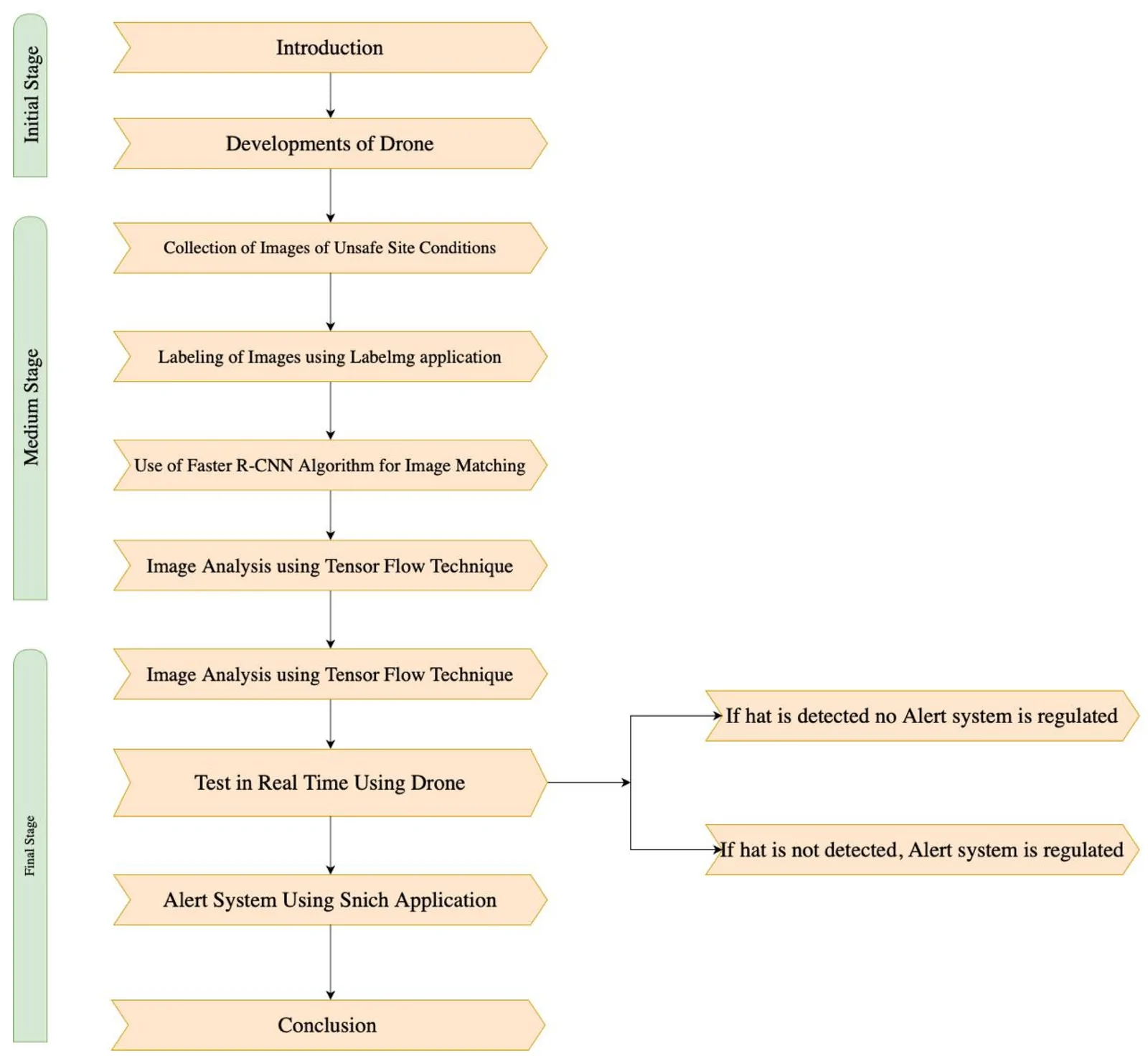
Process of Building UAV for Construction Surveillance.

The use of UAVs fitted with sophisticated object-tracking cameras for overseeing safety compliance in construction settings adheres to a methodical and scientifically sound approach. The UAV is assigned to obtain high-resolution video recordings of the building site. The UAV’s integrated object-tracking cameras are designed for real-time picture identification, using advanced algorithms to differentiate between various items in the area, including personnel and safety equipment, particularly helmets. In contrast to conventional cameras that only capture pictures, these sophisticated systems actively analyze continuous video feeds to detect compliance breaches concerning helmet use. Upon collection of the film, the approach advances to picture preparation. This phase encompasses critical procedures such as normalization, scaling, and data augmentation, which enhance the photos for further analysis. The subsequent step involves feature extraction using deep learning architectures such as Convolutional Neural Networks (CNNs), Faster R-CNN, or R-CNN. These models function by identifying pertinent properties related to helmets, such as form, color, and texture, facilitating precise recognition even under the dynamic conditions typical of construction sites [25]. Their absence limits researchers’ and practitioners’ capacity to reliably reproduce findings. Fluctuations in these factors may result in varying results in helmet detection accuracy, thereby compromising the dependability of the monitoring system. The overall efficacy of the procedure may be questioned when efforts are made to replicate the results in other contexts or with various datasets. Furthermore, the intricacy of the technology requires exact timing for the many operations involved in the detection system. To provide efficient real-time monitoring, the algorithms must function at elevated frame rates, preferably reaching a minimum of 30 frames per second (FPS) [26]. This need is essential for the system to effectively monitor rapid topics and swiftly address safety infractions. Delays in processing time, particularly during feature extraction, categorization, and alarm generation, can undermine the real-time functionality of the monitoring system [27]. While the use of UAVs with object-tracking cameras for helmet identification at construction sites represents a notable progress in safety compliance, the lack of explicitly stated constraints the method’s repeatability. Recognizing and mitigating this restriction, while ensuring appropriate processing time, is crucial for evaluating the efficacy and dependability of this novel safety management strategy. Figure 1 illustrates the process involved in building the UAV for construction surveillance.

### 2.1. UAVs Imaging System for Construction Site Monitoring

The study utilizes the F450 Quadcopter UAV, a robust and versatile unmanned aerial vehicle equipped with an object tracking camera to monitor construction sites. The UAV’s aerial capabilities provide a comprehensive view of the site, enabling it to navigate complex environments and capture high-resolution images and video footage. This approach offers significant advantages over traditional ground-based monitoring systems, particularly in terms of mobility and the ability to access hard-to-reach areas. The object tracking camera embedded in the UAV is specifically designed for real-time monitoring. It captures continuous video streams, which are then analyzed by the tensorflow-based neural network model. The camera’s ability to track moving objects and maintain focus on workers ensures that the system can accurately identify safety compliance throughout the workday. A crucial component of developing an effective object recognition model is the availability of a comprehensive and well-annotated dataset. For this study, images of workers with and without helmets are collected from real-time construction sites. The dataset is divided into two categories: “workers with helmets” and “workers without helmets”. This categorization is essential for training the neural network to recognize and differentiate between the two scenarios. Data collection involves capturing a wide range of images under varying conditions, including different lighting, weather conditions, and worker postures. This diversity ensures that the model can generalize well to new, unseen data. Each image is then labeled using annotation tools, indicating whether the worker is wearing a helmet or not. This labeled dataset serves as the foundation for training the tensorflow-based neural network.

### 2.2. Training the Neural Network Model

Training the neural network involves feeding the labeled dataset into the tensorflow framework, where the CNN learns to recognize the features associated with workers wearing helmets [28]. The training process includes several stages: 1. Data Augmentation: To increase the robustness of the model, data augmentation techniques such as rotation, scaling, and flipping are applied to the training images. This step helps the model learn to recognize helmets under various conditions. 2. Model Architecture: A suitable CNN architecture is designed, typically consisting of multiple convolutional layers for feature extraction, followed by pooling layers to reduce dimensionality, and fully connected layers for classification. The final layer uses a softmax activation function to output the probability of each class (with helmet or without helmet). 3. Loss Function and Optimization: A loss function, such as categorical cross-entropy, is used to measure the difference between the predicted and actual labels. Optimization algorithms like Adam or SGD (Stochastic Gradient Descent) are employed to minimize the loss and improve the model’s accuracy. 4. Training and Validation: The model is trained on the labeled dataset, with a portion reserved for validation. This validation set is used to evaluate the model’s performance and prevent overfitting. Techniques such as dropout and regularization are applied to enhance generalization.

### 2.3. Real-Time Image Processing and Object Detection

**Figure 2:**
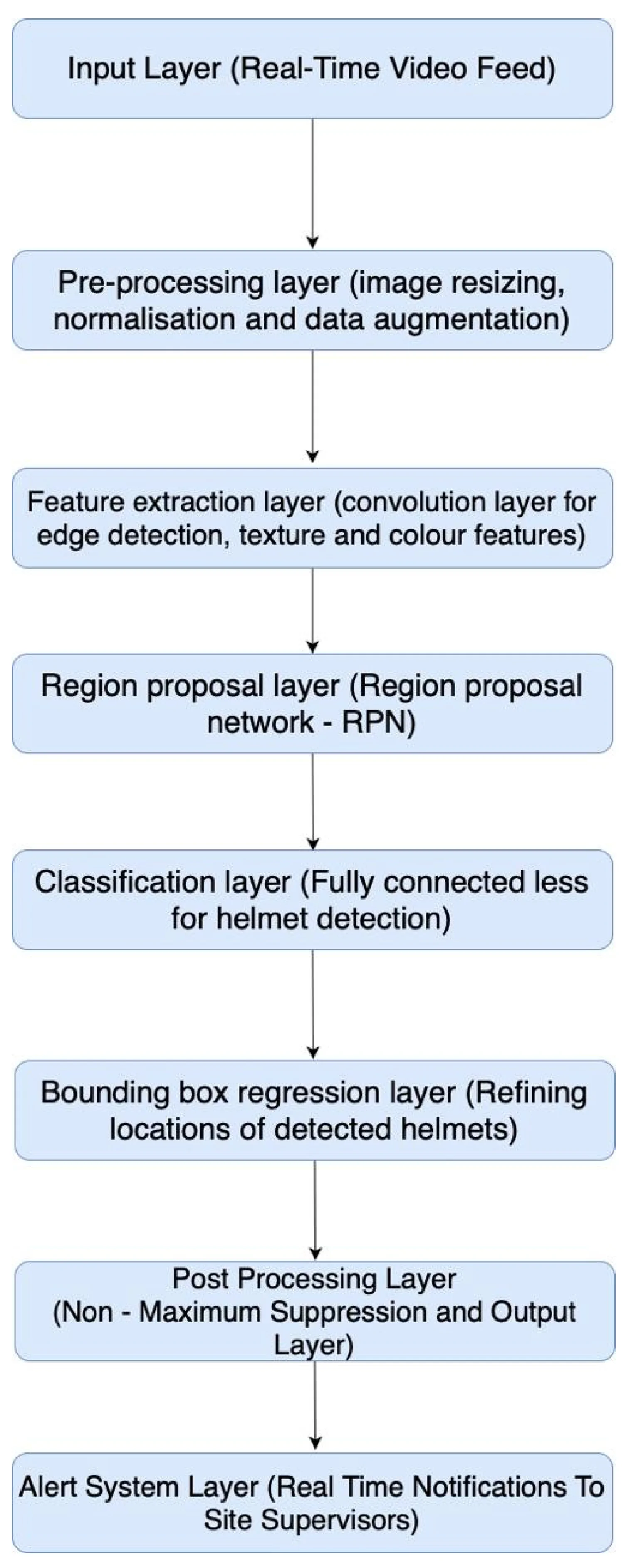
Process of R-CNN based Helmet Identification.

Once the neural network model is trained and validated, it is deployed to work in tandem with the UAV’s object tracking camera. As the UAV patrols the construction site, the camera captures real-time video footage, which is fed into the tensorflow model. The model processes each frame, detecting and classifying workers based on whether they are wearing helmets. The system provides real-time feedback, labeling each detected worker as “with helmet” or “without helmet”. This labeling is displayed on a monitoring screen, allowing site supervisors to quickly identify safety compliance issues. The real-time nature of the system ensures immediate detection of non-compliance, enabling prompt corrective actions to prevent accidents and enhance worker safety. The main goal of this article is to create a prototype model of an Unmanned Aerial Vehicle (UAV) that is particularly built to monitor construction site workers. The purpose of this UAV is to guarantee that workers follow safety regulations and are kept safe from hazardous circumstances. Figure 2 represents the process of helmet identification using the R-CNN approach.

The main objective of the UAV is to assess if workers are wearing helmets, which are an essential component of PPE required to mitigate head accidents at construction sites. The UAV is outfitted with state-of-the-art cameras designed for object tracking, as well as a real-time alarm system that uses image recognition technology to identify workers who are not wearing helmets. Once detected, the UAV may promptly transmit alerts to the supervisors at the site, motivating appropriate actions to address safety violations. This strategy not only improves the effectiveness of safety compliance monitoring but also decreases the need for manual inspections by supervisors, resulting in a more efficient and less labor-intensive procedure. The objective of the prototype is to incorporate these technologies into a lightweight and modular UAV that can be quickly deployed and easily maintained. This will provide a strong option for enhancing worker safety on construction sites. The suggested architecture for a construction safety monitoring system using tensorflow and UAV technology aims to improve worker safety via real-time picture processing. The design starts with the input layer, which receives continuous video streams from UAVs outfitted with high-resolution cameras monitoring the building site. The acquired pictures are further processed in the Preprocessing Layer, where they are resized and normalized to achieve consistent pixel values, in addition to using data augmentation methods such as rotation and brightness modifications to improve the model’s resilience to diverse environments. Subsequent to preprocessing, the Feature Extraction Layer utilizes convolutional layers to derive pertinent features, including edges, textures, and color patterns related to helmets and other PPE. The Region Proposal Layer subsequently suggests prospective areas where helmets may be placed, creating bounding boxes around these candidates for more examination. In the Classification Layer, fully linked layers categorize the suggested areas as either “with helmet” or “without helmet”, enabling the model to accurately differentiate between compliant and non-compliant personnel. Thereafter, the Bounding Box Regression Layer adjusts the coordinates of the bounding boxes to guarantee precise localization of identified helmets inside the photos. The Post-Processing Layer employs methods like Non-Maximum Suppression (NMS) to remove redundant bounding boxes, preserving just the most reliable predictions. The Alert System Layer is tasked with dispatching real-time messages to site supervisors upon the detection of non-compliance, hence furnishing essential information for timely action. This methodical method, shown in Figure 1, demonstrates the tiered architecture and emphasizes the contribution of each component to the overarching objective of enhancing safety compliance on construction sites using sophisticated image recognition algorithms. The system utilizes tensorflow and UAV technology to proactively detect safety infractions, enabling prompt interventions and improving adherence to safety requirements.

### 2.4. UAV Peripheral Interface

**Figure 3:**
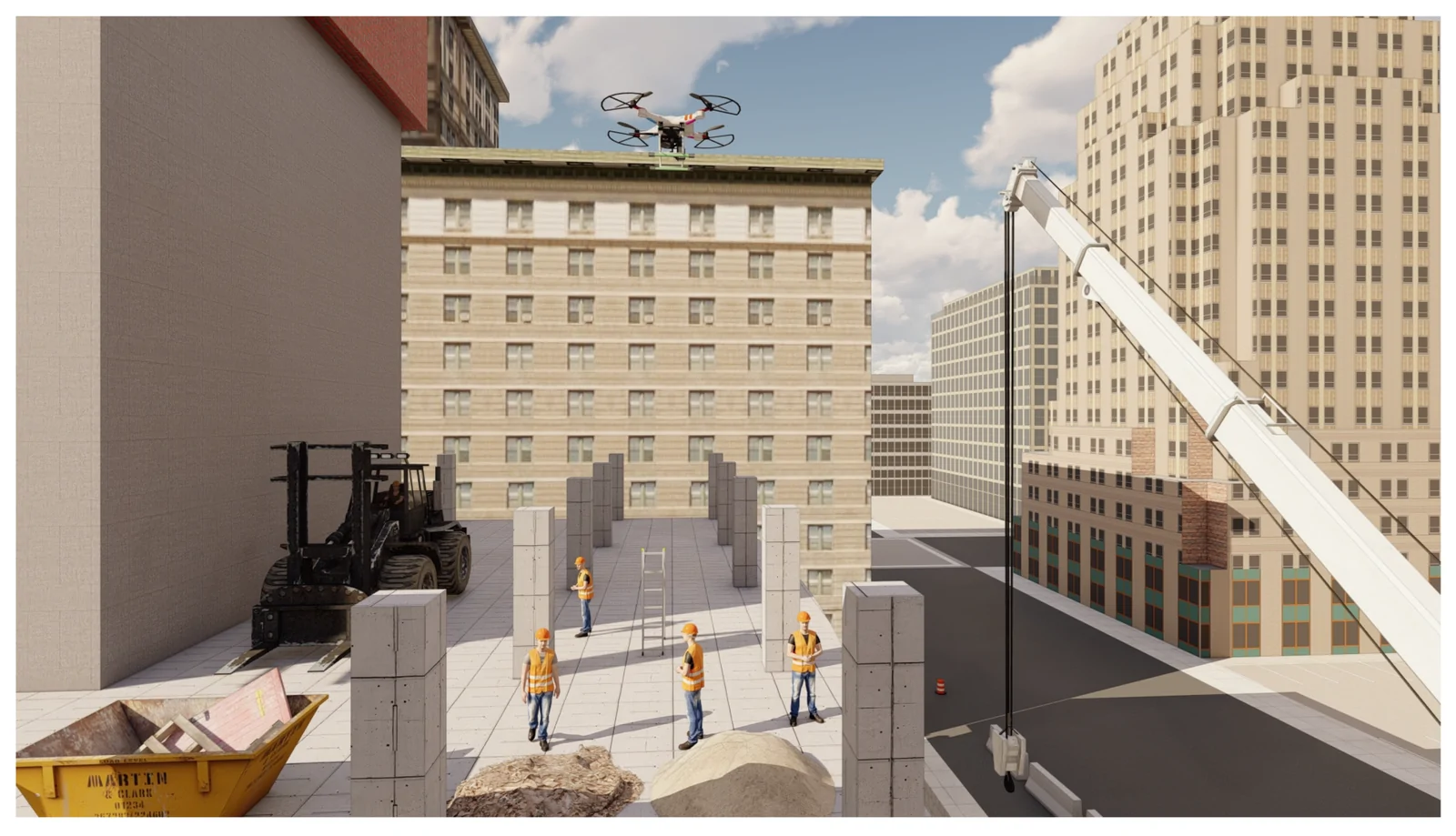
Layout of the Construction Sit Monitoring Using UAV.

The sky stars’ flight controller is used to construct a UAV platform that integrates multiple frames. F4 F405 HD is our current quadrotor frame, and the F4 processor powers this board. The Sky Stars F405 Flight Controller has a dedicated 10V output for the DJI air unit and a typical analog video on-screen display (OSD). This board also includes a 640 × 480 optic flow camera. The Sky Star flight board has six universal asynchronous receiver-transmitter (UARTS) connectors. That connects various interfaces for electronic speed controllers (ESC), remote controls, and GPS module connectors that output a micro-air vehicle communication protocol to ensure compatibility with all fixed-wing and rotor UAV frames while conserving IO ports (mavlink). The Layout of the Construction Sit Monitoring Using UAV is represented in Figure 3. 2 GB of synchronous dynamic random access memory and 32 GB of flash memory are available on the Sky Star flight board for Trusty and other applications. Qualcomm previously provided peripheral drivers and UART apis, so getting data from the IMU sensor and the other two sensors in this system will be straightforward. Object detection is based on deep learning. Automatic hat detection is a subset of construction-related object detection. Visual object recognition is ineffective if an image lacks the desired elements. HOG (Histograms of oriented gradients) was previously used for pedestrian identification by Dalal et al. In 2005 [29]. The DPM (deformable part-based model) and larger HOG filters have since been created to improve HOG-based approaches. The difficulty in training and the need for specifically developed learning techniques have typically questioned these approaches [30]. The difficulties in object detection, recognition, and tracking in a hazy foundation are since, in reality, the foundation is obscure. The term “hazy foundation” refers to any foundation that tends to shift in light and is comprised of many different, subjective components. The UAV system is versatile and controllable in operation; it can take off and land based on instructions from a radio transmitter and can be remotely controlled by a wireless electric remote controller or an airborne computer.

### 2.5. UAV-Based Image Recognition System

**Figure 4:**
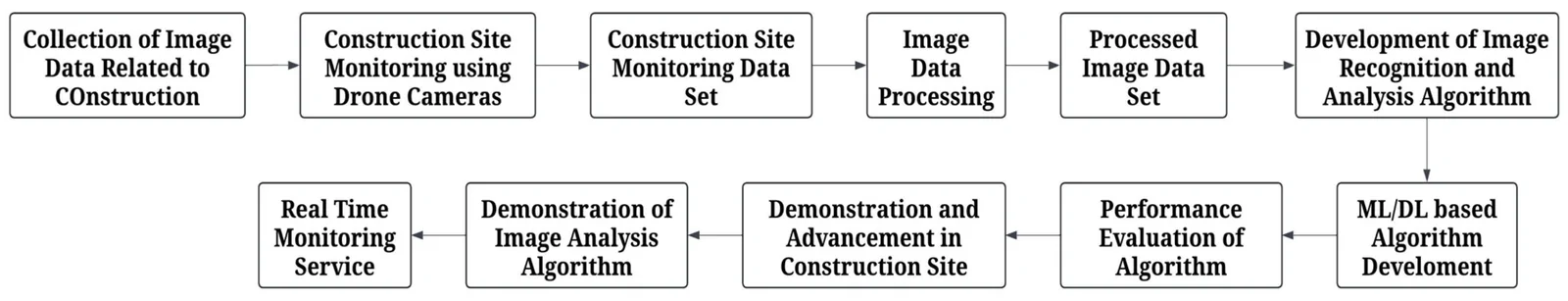
Process of UAV-based image recognition analysis technology for worker safety.

The integration of advanced image recognition technology, particularly through the utilization of drones, is pivotal for enhancing worker safety in modern construction environments. The process begins with the systematic collection of image data relevant to the construction site, where drones equipped with high-resolution cameras capture visual information. Typically, these cameras produce images with a resolution of 1920 × 1080 pixels (HD) or higher, ensuring adequate detail for effective analysis. The depth of the images, often represented in 24-bit RGB color space, allows for precise differentiation of objects based on color and texture. The type of images collected includes both still frames and video streams, enabling comprehensive monitoring of worker activities. Once collected, this visual data are organized into a structured dataset specifically designed for construction site monitoring. The dataset undergoes rigorous image preprocessing, which may include resizing to standard dimensions (e.g., 416 × 416 pixels for faster processing), normalization to scale pixel values between 0 and 1, and augmentation techniques such as rotation, flipping, and cropping. These steps enhance the dataset’s diversity and robustness, preparing it for further analysis. At the core of this technological advancement is the development of algorithms for image recognition and analysis, leveraging the tensorflow framework to implement advanced machine learning techniques. tensorflow facilitates the creation of multiple CNN architectures that are essential for determining compliance with safety protocols, such as helmet usage among workers. Among these models, Faster R-CNN (Region-Based Convolutional Neural Network) stands out due to its efficiency in real-time object recognition [31]. By utilizing a Region Proposal Network (RPN), Faster R-CNN streamlines the identification process, enabling rapid detection of workers and their adherence to helmet regulations. Moreover, the system also incorporates RCNN (Regions with CNN) methodologies to further enhance detection accuracy. This dual approach ensures that the system effectively differentiates between compliant workers wearing helmets and those violating safety protocols. The algorithm development process involves meticulous training and validation phases to guarantee accuracy and reliability. The parameters such as learning rate, batch size, and number of epochs are optimized during training to enhance model performance; however, these parameters must be clearly defined to ensure reproducibility in different contexts. One of the key advantages of this method is its ability to operate under various site conditions, such as differing lighting scenarios, weather variations, and construction site layouts. The preprocessing and augmentation techniques contribute to the model’s robustness, enabling it to maintain high accuracy in challenging environments. For instance, the use of adaptive thresholding in image segmentation can help mitigate the effects of shadows or glare, while training on diverse datasets ensures that the model can generalize well across different conditions. Once validated, the algorithms are deployed in real-world construction settings, marking the transition to practical application and continuous improvement. This proactive approach allows for the real-time monitoring of safety compliance, providing immediate feedback and notifications to site supervisors when violations are detected. Such responsiveness not only enhances enforcement of safety protocols but also significantly reduces the risk of accidents related to helmet non-compliance. By effectively implementing these advanced image analysis techniques, the system facilitates the establishment of a real-time monitoring service that consistently supervises worker safety on-site. Figure 4 illustrates the comprehensive workflow of drone-based technology for image recognition and analysis, highlighting the integration of tensorflow and CNN models, namely Faster R-CNN and RCNN, to significantly improve worker safety in construction environments.

### 2.6. Data Postprocessing Process

**Figure 5:**
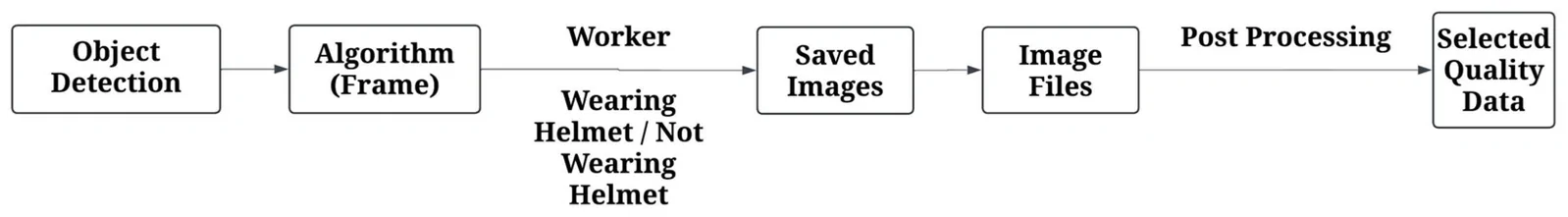
Procedure for collecting construction site data and postprocessing.

The rapid integration of UAVs in the construction sector has transformed safety, surveying, quality oversight, and maintenance evaluation. UAVs are essential in mitigating accidents associated with falls from heights or being impacted by falling items by ensuring adherence to safety measures among workers. This project aims to use UAV technology to improve labor safety by monitoring the utilization of PPE, primarily helmets, among construction workers. The created UAV system utilizes tensorflow methodologies and an alert mechanism to detect and identify workers lacking helmet use. The UAV uses the high-precision, high-speed Faster R-CNN algorithm to correctly recognize construction workers, both helmeted and unhelmeted, in real time under diverse site circumstances. This proactive strategy guarantees prompt input and response, substantially decreasing the likelihood of injuries and deaths. Worker safety is essential on modern construction sites, with helmet use being a vital safety measure. The drone technology automates compliance with helmet rules using object detection, while the UAV records real-time video footage of the location. An algorithm evaluates these streams to identify workers and verify helmet use, categorizing each frame as “Wearing Helmet” or “Not Wearing Helmet”. The findings are graphically recorded, with photos processed to keep only high-quality frames that exhibit accurate detection results. This finally yields a curated set of high-quality data that delivers dependable insights on helmet use, aiding site managers in effectively implementing safety regulations. Figure 5 represents the procedure for the data collection and postprocessing.

To establish the construction worker image dataset was collected. More than 500 images were gathered from two on-going construction sites; “Vendhar Square”, SRM Institute of Science and Technology, Kattankulathur, Chennai, Tamil Nadu; and “Kharkai Barrage Constructions”, Adityapur, Jharkhand. The images were collected under various conditions and occlusions to create a comprehensive dataset. It refers to retrieving our dataset from the data warehouse. In this approach, the datasets will be stored in a git repository, and multiple datasets will be integrated to generate a bigger knowledge base. No open-source dataset was available for this implementation at the time of this study. Images recorded at ongoing construction projects and construction fields during surveying and monitoring were used to create the dataset. Photos of laborers on construction sites were categorized as “with a hat” or “without a hat”. Python can read XML files with annotations saved in Pascal VOC format because they were held in this format.

### 2.7. Helmet Monitoring Model

**Figure 6:**
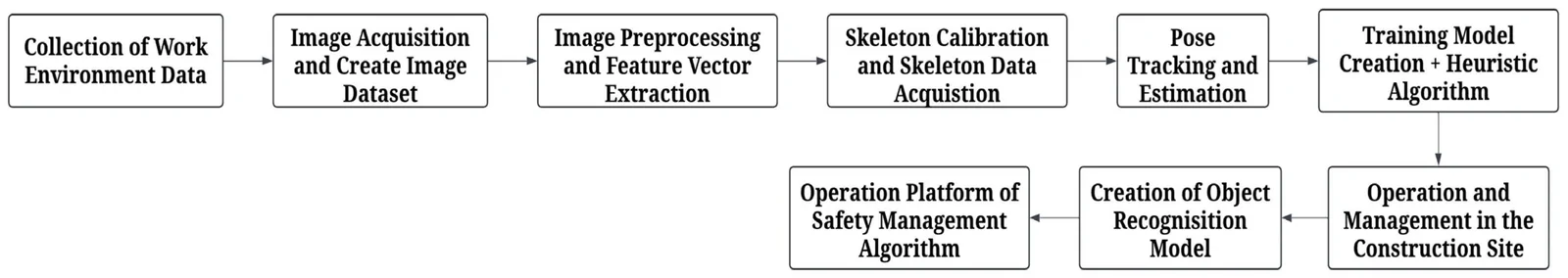
Overall Helmet monitoring model.

Developing sophisticated safety management systems for construction sites requires a methodical approach to guaranteeing the well-being of workers. The process starts with the accumulation of data pertaining to the work environment, whereby meticulous details about the location are collected to construct a complete monitoring system. Subsequently, the process of obtaining photos and assembling an image dataset takes place, whereby photographs of the location are acquired and consolidated, serving as the foundation for further analysis. Next, the process advances to image preprocessing and feature vector extraction, where the pictures are processed and important characteristics are retrieved to improve the accuracy of the analysis. As seen in Figure 6, the development model for monitoring workers’ helmet use involves two further processes: skeleton calibration and skeleton data collection. These steps enable the system to accurately acquire and calibrate skeletal data, enabling precise tracking of workers’ movements. This results in posture tracking and estimation, which allows for the real-time monitoring of workers’ locations and movements on the site. The collected data are next used in the training and model generation stage, when machine learning models, augmented by heuristic algorithms, are constructed to identify items and behaviors associated with worker safety. Once the models are developed, they are included in a safety management algorithm operating platform that is specifically built to oversee the site. Ultimately, the system transitions into the operation and management phase at the construction site, where it is actively used to guarantee continuous worker safety. This methodical approach not only improves the ability to monitor in real-time but also establishes a strong foundation for detecting and reducing possible safety hazards at building sites.

**Figure 7:**
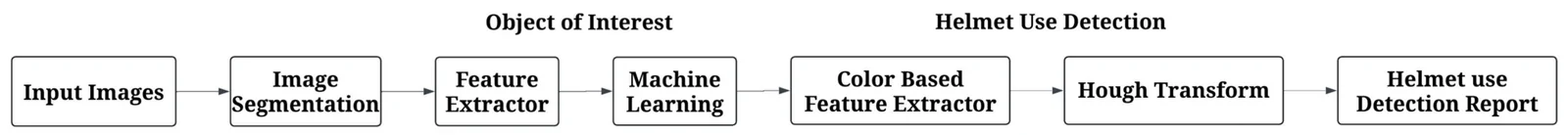
Helmet Detection and Alter System.

The experimental component of the paper is vital for corroborating the efficacy of the suggested helmet identification system; nevertheless, it omits crucial metrics such as mean average precision (map), which is critical in the domain of object detection. Map offers a thorough assessment of the model’s efficacy by computing accuracy and recall at various intersection over union (IOU) criteria. This statistic enables a detailed assessment of the model’s efficacy in identifying helmets across many settings, including comparisons with other leading object identification techniques. Furthermore, while the discourse on real-time monitoring is essential, the lack of frames per second (FPS) data undermines the transparency of the system’s operating capabilities. FPS measures the number of frames the system can process per second, directly affecting its use in dynamic real-world building settings. A system that processes video at 30 FPS can provide prompt notifications to site managers about safety compliance, which is essential for maintaining ongoing surveillance of worker safety. Figure 6 delineates the systematic technique for helmet identification in construction monitoring, especially tailored for UAV (Unmanned Aerial Vehicle) applications. The procedure begins with photographs obtained by drones, which undergo image segmentation processes. Segmentation partitions the picture into discrete parts, facilitating the separation of areas of interest, such as on-site personnel. Subsequent to segmentation, the system evaluates these areas to extract essential characteristics like form, color, and texture. This feature extraction utilizes sophisticated machine learning models, perhaps using frameworks like tensorflow and deep learning architectures such as Convolutional Neural Networks (CNNs), Faster R-CNN, or R-CNN [32]. Each model is essential for successfully identifying helmets by learning to discern patterns related to helmet forms and colors from a training dataset. A color-based feature extractor is used to focus on certain color properties relevant to helmets. This extractor uses color histograms or color moment characteristics to identify helmet-like hues in the segmented areas. The approach may also include the Hough Transform algorithm, which is proficient at detecting geometric patterns, particularly the circular shape typical of helmets [33]. This phase improves identification capabilities by enabling the algorithm to exclude non-helmet items and concentrate primarily on recognizing helmets via their distinctive geometric and color characteristics. This method culminates in a detailed report that records the detection findings, specifying whether helmet compliance was noted in the examined photographs. This pipeline’s automated design, seen in Figure 7, facilitates the monitoring of safety compliance on construction sites, guaranteeing that workers follow helmet use standards without requiring substantial human supervision. By optimizing this process via automation and using advanced algorithms, the system optimizes safety standards and boosts operational efficiency in potentially hazardous work conditions. The ongoing development and integration of sophisticated detection systems are crucial for enhancing safety measures in the construction sector, offering prompt warnings and fostering a safer working environment for all staff engaged. Integrating measurements such as map and FPS in next studies will provide a more comprehensive evaluation of the system’s performance and real-time monitoring capabilities.

## 3. Implementation

Tensorflow, especially when used with GPU acceleration, functions as a robust open-source software suite intended for high-performance numerical modeling. Its design facilitates easy deployment of computations across several platforms, making it an indispensable asset in machine learning and deep learning domains. The essence of tensorflow’s capabilities is in its versatile numerical calculation engine, applicable across several scientific disciplines. In image analysis, tensorflow is especially adept at training CNN to identify and categorize items, such as safety helmets, coats, and cars, which is essential for maintaining compliance with safety rules in hazardous work conditions [34]. The procedure starts with data gathering, whereby photographs of on-site personnel are obtained, emphasizing certain categories pertinent to workplace safety. This information is then annotated with labels denoting the presence or absence of safety gear, creating a thorough training set. The training procedure entails inputting the labeled information into the CNN, enabling the model to discern patterns and characteristics linked to each class. The network iteratively modifies its weights using backpropagation and gradient descent methods to reduce classification mistakes. This enables the model to comprehend several qualities, like color, form, and texture, that differentiate helmets from other items in the photos. Tensorflow offers comprehensive tools for picture preparation to improve model efficacy. Methods including normalization, scaling, and data augmentation are used to guarantee that the model generalizes well across various lighting circumstances and viewpoints found in real-world situations. Data augmentation increases diversity in training data via random changes, therefore mitigating overfitting and enhancing the model’s resilience. Upon completion of training and validation of the CNN, the deployment step commences, during which the model is incorporated into a real-time image processing system. This arrangement allows the continuous oversight of on-site personnel to guarantee adherence to safety regulations. The tensorflow-based system can rapidly detect workers violating safety requirements, such as the absence of helmets, by analyzing live video feeds or photos from UAVs or fixed cameras and promptly notified site managers. Moreover, tensorflow’s adaptability enables the incorporation of sophisticated methodologies like transfer learning, whereby pre-trained models on extensive datasets may be refined for particular applications. This method expedites the training process and improves performance, especially when the quantity of accessible labeled data are limited. The capacity to use robust GPU resources for computing markedly accelerates training and inference durations, facilitating efficient system operation in dynamic building settings. Tensorflow functions as a fundamental instrument for picture analysis, especially in safety compliance applications inside construction sites.

**Figure 8:**
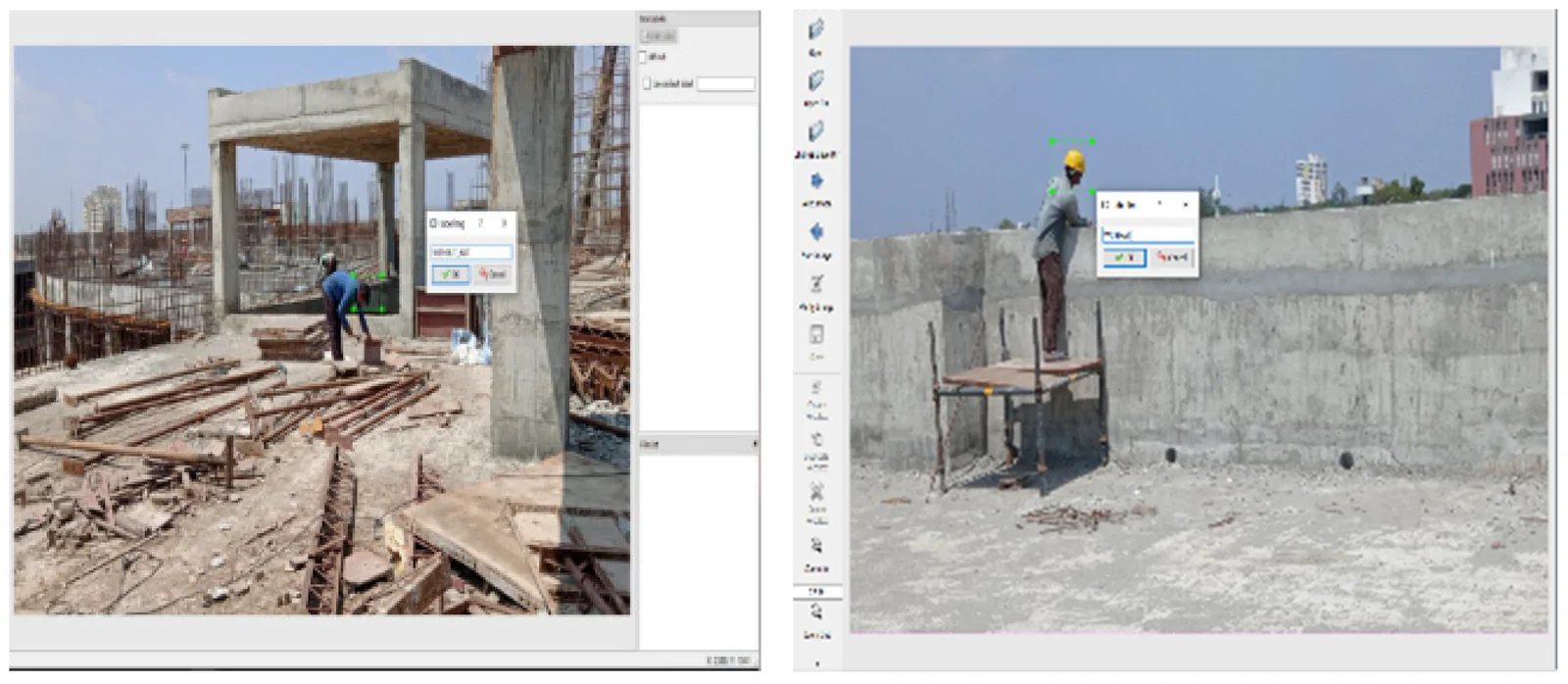

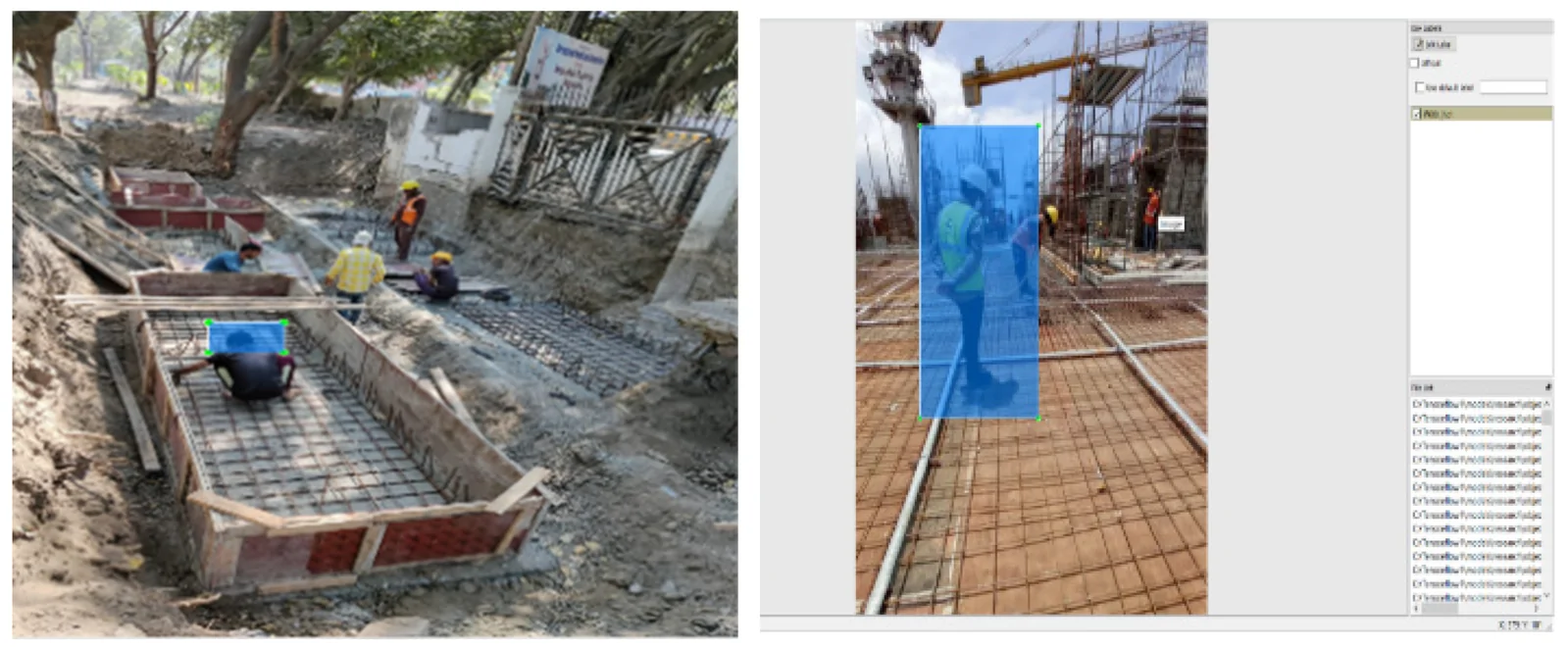
Initial Stage Image frames stepwise labeling.

Its capacity to educate CNNs for identifying critical safety equipment not only bolsters worker safety but also optimizes monitoring procedures, thus fostering a safer workplace. Tensorflow enhances proactive safety management in hazardous settings by optimizing data collection, labeling, training, and real-time deployment procedures. Figure 8 represents the real-time captured image at the construction site.

### 3.1. Labeling of Images

Label the desired objects in each image after gathering all of the images. Labeling is the annotation tool used in this experiment. The annotation tool can recognize general locations, activities, products, and accessories. The images that have been gathered are labelled here. A box is created around each object in each image in the directory. Labeling 7 makes an an.xml file for testing and training. Figure 8 shows the labeling of images. A training set and a testing set were derived from the complete dataset to train the data correctly. In this case, the Fater RCNN algorithm is employed. The RCNN-Inception-V2 model training got much faster after the value dropped below 0.8. It will take roughly 50,000 steps or 3 h to train the model to a consistent loss of less than 0.05. Previously, it was stated that there are two unique images of labor. The several layers indicated in “Model conversion” are used to train this. Using the Faster R-CNN model, this convolution is accomplished.

### 3.2. Flow of the RCNN Model

**Figure 9:**
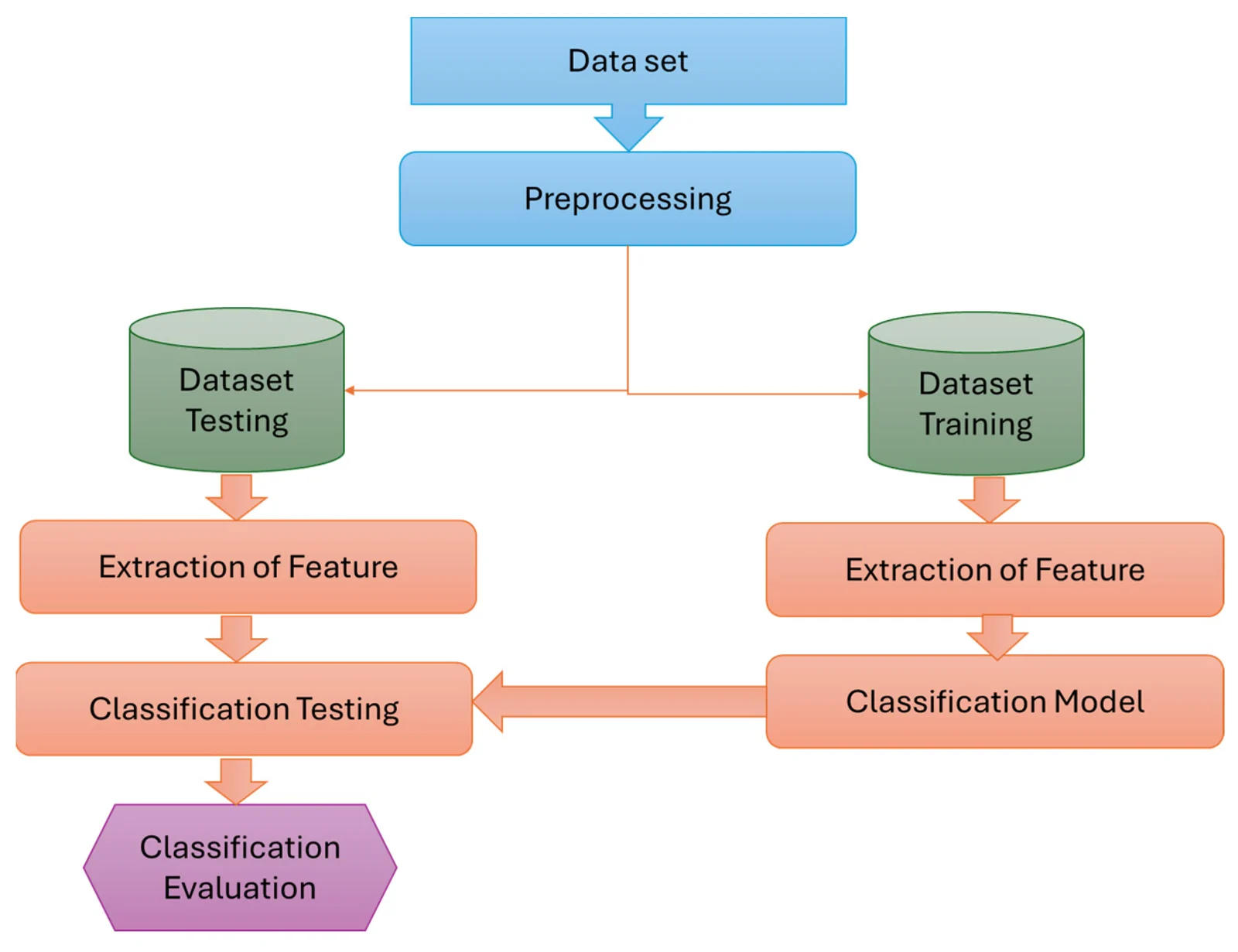
General pipeline of the model.

Figure 9 shown below shows the pipeline of the model. Extraction of features is performed in two parts of the dataset, as stated previously. Once trained, the model is used as a basis for the final algorithm (Faster RCNN), and analysis is followed by labeling of images, formation of bounding boxes in images, and preparation of data explained below. The Faster R-CNN object identification algorithm was introduced by Ren et al. in 2015 [35]. A More Rapid Regional Proposal Network is a new venture for CNN that can generate high-quality region proposals and have several ideas to detect and categorize things that share RPN’s complete image convolutional characteristics [36]. Faster—The RCNN-Inception model is slower but more accurate. Keeping accuracy in mind, Faster RCNN was chosen over other algorithms because these models allow faster detection but lower accuracy. Images need to form bounding boxes for object detection. To construct the discovery model, we must refer to many factors, such as each image’s height, breadth, and class name. Enclosing bound x-min, max values, y-min, and max values are also included in the dataset. tensorflow is a robust framework for defining, customizing, and designing various models. Tensorflow is a free, open-source programming library that can be used in various situations for data flow and differentiable programming. It is a math library for AI applications that follows logical reasoning. The pre-loaded dataset of 100,000 photos has been preprocessed by the tensorflow Inception CNN model. As a result, tensorflow plays a critical role in the data collection process. After that, the construction workers’ image dataset should be developed. There is no off-the-shelf dataset available. An appropriate dataset was created to teach the Faster R-CNN to detect helmet and non-helmet behavior. Images were collected from several sites and ongoing construction projects to create a rich dataset. More than 500 images were gathered by visiting various sites to create the construction worker image dataset. The images were collected from multiple sites to create a comprehensive dataset. The pictures contained in the afternoon were clear and noise-free. The training set comprised 500 images drawn from this dataset. A quicker R-CNN hat model was created using the combined data. With the remaining images, a testing dataset was created.

R-CNN and Faster R-CNN are both significant models in object identification, although they embody different methodologies within the same general framework. R-CNN was among the first models to successfully use deep learning for object identification. The fundamental concept of R-CNN is using selective search to generate candidate object areas in an image, then classed by a CNN. The procedure starts with the development of around 2000 area proposals from an image, then followed by feature extraction from each proposal using a CNN. Subsequently, each area is categorized using a Support Vector Machine (SVM), and bounding box regression is used to enhance the precision of the anticipated item positions. Although R-CNN significantly enhanced object detection efficacy, its dependence on selective search for region proposal creation rendered it computationally intensive and slower since each area necessitates unique processing via the CNN. Faster R-CNN, as indicated by its nomenclature, is an enhancement of R-CNN that rectifies its deficiencies. The principal advancement in Faster R-CNN is the implementation of a Region Proposal Network (RPN) that produces region proposals more efficiently and cohesively. Faster R-CNN utilizes the RPN to create region suggestions directly from the feature maps produced by the CNN, rather than relying on selective search. This optimizes the object detection process, enabling the model to propose and categorize areas inside a singular, cohesive framework. The RPN shares convolutional layers with the object detection network, enhancing both processing speed and the accuracy of recommended areas since these suggestions are derived from characteristics directly pertinent to the classification objective. Faster R-CNN generally surpasses R-CNN in both velocity and precision. Faster R-CNN attains real-time processing capabilities by omitting the distinct region proposal phase, rendering it more appropriate for situations necessitating rapid decision-making, such as building site safety monitoring. The efficacy of Faster R-CNN in identifying helmet use among workers enables real-time analysis of video feeds from UAVs, delivering prompt feedback to verify adherence to safety rules [37]. R-CNN and Faster R-CNN are both robust object detection models, although they fundamentally vary in their methodologies for region proposal creation and processing velocity. R-CNN depends on selective search, resulting in diminished performance, whereas Faster R-CNN utilizes an integrated RPN that markedly improves efficiency and accuracy, rendering it more appropriate for real-time applications such as worker safety monitoring in construction settings.

The Faster-RCNN algorithm underwent a particular evaluation to determine its effectiveness in recognizing whether construction workers are wearing helmets, utilizing footage acquired by drones. The model underwent training using the following parameters: a training batch size of 1, a testing batch size of 1, 4 classes, a learning rate of 0.005, and 50 epochs.

## 4. Results and Discussion

**Figure 10:**
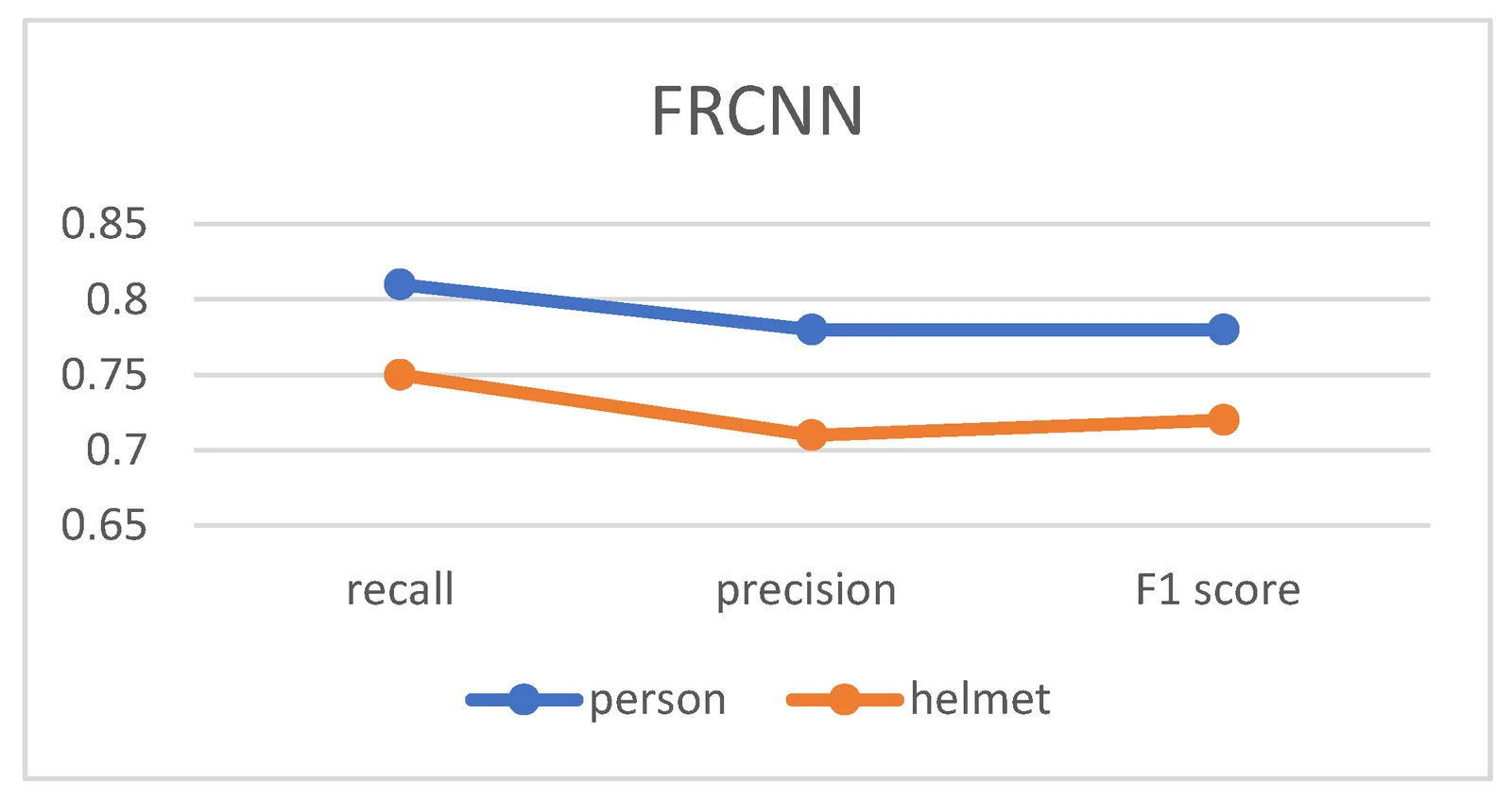
FRCNN Results.

Figure 10 depicts the identification of helmets worn by construction workers using the Faster-RCNN model. The graphic illustrates the algorithm’s process of detecting and categorizing whether workers are wearing helmets or not. The model attained the following metrics for helmet detection: a recall of 0.75, a precision of 0.71, and an F1 score of 0.72. The model achieved a recall of 0.81, an accuracy of 0.78, and an F1 score of 0.78 for recognizing the presence of individuals. These metrics demonstrate the model’s efficacy in differentiating between individuals who are wearing helmets and those who are not, as well as reliably identifying individuals. The model’s trustworthiness is further emphasized by the overall accuracy of 0.75, which encompasses the performance across all classes. Nevertheless, when specifically considering helmets, the model exhibited robust performance even in intricate situations. The scenarios included visual problems such as occlusions, where helmets may be partly concealed, or small differences in appearance, such as various helmet colors or angles. Despite the challenges encountered, the model regularly and reliably identified whether workers were wearing helmets, demonstrating its efficacy in monitoring safety compliance using drone-based surveillance.

The Faster RCNN model was tested on a wide variety of images. Following is a breakdown of the accuracy in various conditions. The accuracy measurements were employed primarily to identify workers who were not wearing helmets.

The accuracy measures were chosen with the primary goal of detecting individual employees without helmets in sight. The final classifier was split into two groups: employees without helmets and everyone else. Precision is the first metric, and it is widely used in pattern recognition evaluation. To understand the notion of precision, we must first explain the precision of True Positive (TF), False Negative (FN) and False Positive (FP). In this case, the number of workers who did not wear helmets and whose test results were accurate is shown by the letter TP. No headgear was found on FP objects, but the results are false. Although there are FN employees, there are errors in the results of the tests. A description of TP, FP, and FN can be found in Table 1. The ratio of TP to TP + FP is used to measure detection accuracy and is called precision (1). Based on the approach, the number of workers detected as not wearing a helmet is TP + FP. The recall is calculated by dividing the TP value by the TP + FN value (3). Workers that do not wear helmets are counted as TP + FN. Many workers are overlooked due to their absence of headgear by the system, which is known as the “miss rate”, which is the inverse of recall (2). In comparison to previous approaches, this one has a lot faster computation speed because it is run on a graphics processing unit (GPU). Faster RCNN needs to watch videos from the work site to prove that it can achieve the constraints in real time. The speed of Faster RCNN refers to time consumed by a complete worker without helmet detection for one image.

Accuracy = TP/(TP + FP)(1)

Missrate = 1 − Recall = FN/(TP + FN)(2)

Recal = TP/(TP + FN)(3)

Table 2 describes the various conditions and information on weather collected while capturing images on the construction site. Natural conditions can significantly impact construction sites because they are mostly exposed to the outside environment. As a result, the quality of the surveillance video and images captured is affected by weather changes. In the second row of Table 2, we see the four most prevalent weather conditions: sunny, cloudy, rain, and drizzle. Table 2 shows that the model’s robustness in varied weather circumstances (misty rain and hazy conditions) has a low effect on detection performance, with precision and recall rate staying stable in these settings. However, the results were better on sunny and overcast days. Positions taken by workers in the construction industry vary based on the job, the mechanical tools they employ, and even where they work. Standing is the most common posture for pedestrian detection deep learning. Standing, bending, squatting, and sitting are four common construction worker postures. This test used a dataset of 500 image frames. Here image frames in which workers are wearing helmets for the convenience of metrics are chosen. It is common for construction sites to be full of personnel, machinery, and materials. In far-field surveillance video, equipment, supplies, and other workers frequently obstruct workers. As a result, many workers featured in the videos are no longer present. To assess the impact of occlusions, four categories of occlusion are: “whole body visible”, “upper body visible”, “head visible”, and “section of the head”. Automatic helmet spotting is a subset of object detection used in construction.

**Figure 11:**
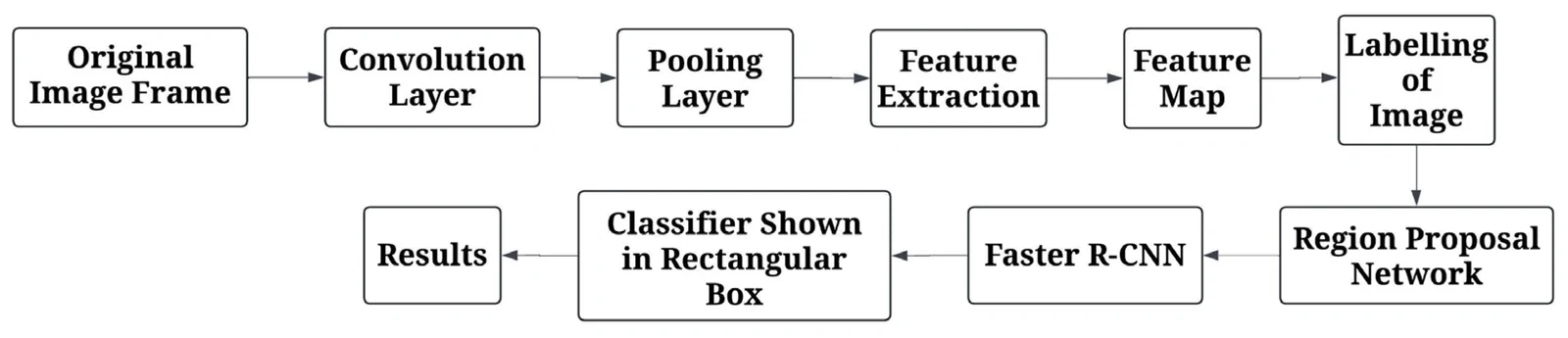
Framework of Proposed systems.

In visual object detection, the goal is to determine whether or not an image contains one or more objects from the class of interest. CNN is a well-known approach for image categorization in deep learning (CNN). Here, tensorflow is used to evaluate image recognition in this study. Due to tensorflow, it could identify the test image 99 percent of the time correctly. Windows 10, Python 2.7, tensorflow GPU v1.5, and the Labeling application were used in this study’s system configuration. Figure 11 shows the framework of the proposed system. Once the images have been labeled, it is time to create the tensorflow Records that will be used to serve the tensorflow training and testing models. With tensorflow-GPU v1.5, the initial version for the training and testing of images is completed. The training will be started by tensorflow. Before the real training begins, the startup process can take up to 30 s. The loss is reported at each stage of training. It will begin high and gradually decrease as training proceeds. Object detection is a strong suit for the deep CNN approach, as demonstrated in the proposed framework, and much research has gone into improving it. Under several extractions, the original image extracts. R-CNN architecture, developed by Girshick and colleagues, produces 500 bounding boxes from a photo and uses a CNN technique to extract the bounding box features [38]. Girshick proposes using only one feature extraction. Fast R-CNN extracts the image’s bounding box features and inserts them into a pooling layer for the ROI (region of interest). Ren et al. respond with their enhanced Faster R-CNN, which uses Region Proposal Networks (RPNs) rather than selective search to acquire the bounding boxes.

Faster R-CNN was chosen over alternative algorithms for picture detection for site monitoring in construction contexts due to the necessity for quicker detection speed. PPE compliance on construction sites relies heavily on workers being identified in real time as wearing said PPE. Faster R-CNN’s superior object detection skills allow it to precisely identify and categorize photos, including workers. The system then flags and emphasizes worker instances according to their PPE compliance status. This clearly separates those who are following safety requirements by wearing PPE from those who are not. In addition to improving safety monitoring, this function helps streamline operations related to supervision and compliance management, making for a less dangerous workplace overall. Labels the recognized objects, making it simpler for users to understand the result and act accordingly. This picture representation improves tasks like monitoring PPE compliance and safety assessment in different environments. In order to ensure that individuals are in compliance with visual safety rules, these representations can be fitted with the proper PPE, such as helmets, goggles, masks, or gloves. Automatic alerts can be set off by the system if an employee is not wearing their PPE, and from there, higher-ups can take action, such as fining or warning the offender. This method is useful for enforcing safety rules and providing a non-intrusive, effective method of checking workers’ use of PPE. Safety monitoring and incident prevention greatly benefit from documentation in the form of images. It is critical that workers and safety personnel prioritize precise and full documentation of safety-related issues through visual media, allowing management to take fast corrective actions and install relevant safety measures. By being proactive with visual documentation, the construction sector may greatly improve its safety measures and create a more secure workplace for its employees.

**Figure 12:**
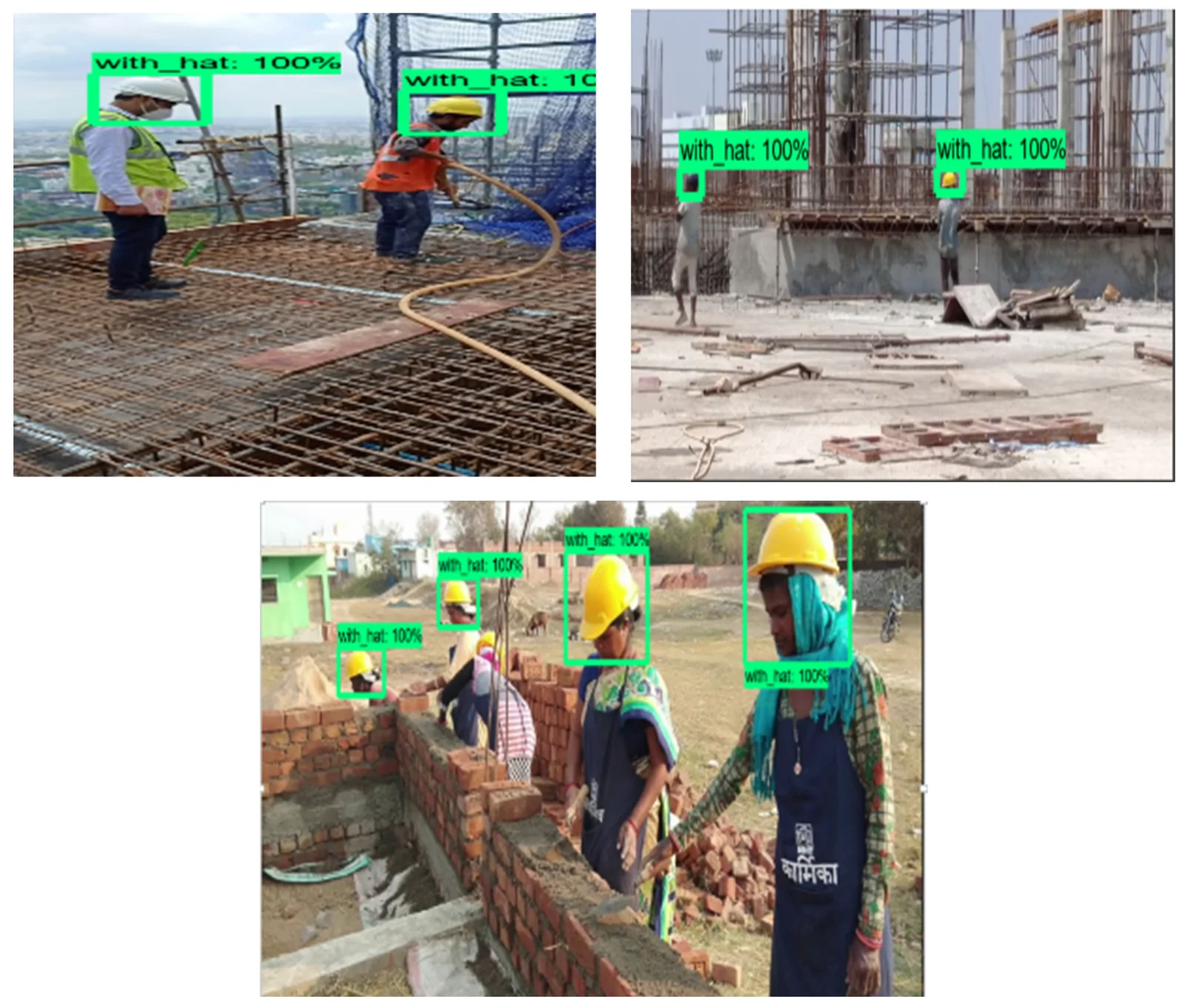
Image frame examples of workers recognized with Helmets.

**Figure 13:**
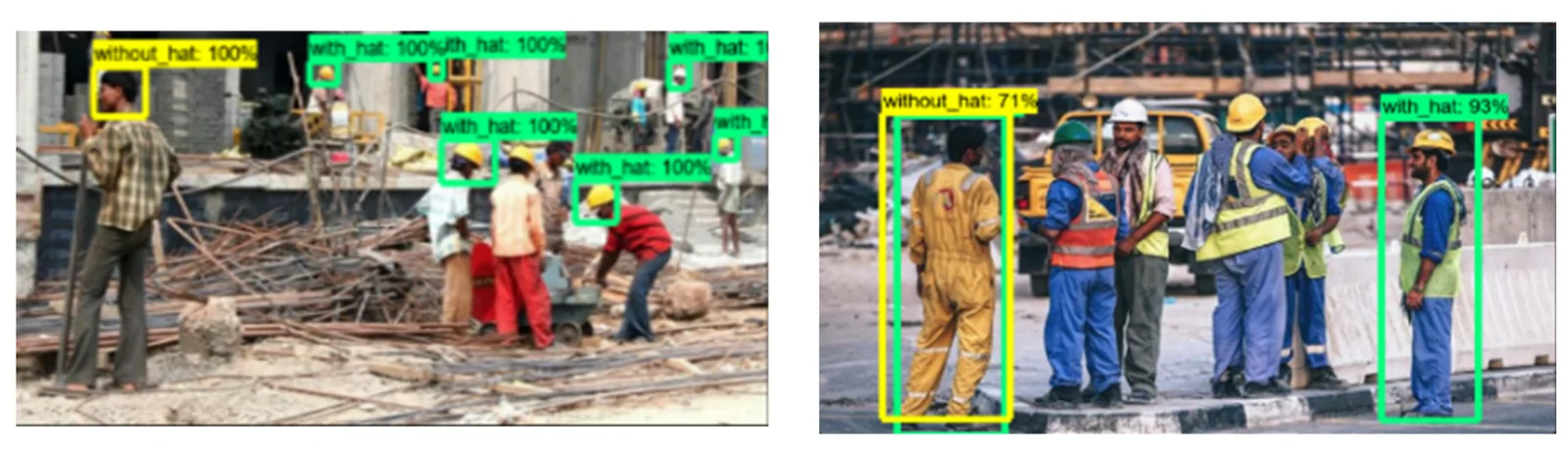
Image frame example of workers recognized without Helmets.

Figure 12 and Figure 13 show the image of workers recognized for object detection of workers without helmets using tensor flow. Construction workers’ postures include standing, bending, crouching, and sitting, as opposed to the four basic poses used by pedestrian recognition software.

**Figure 14:**
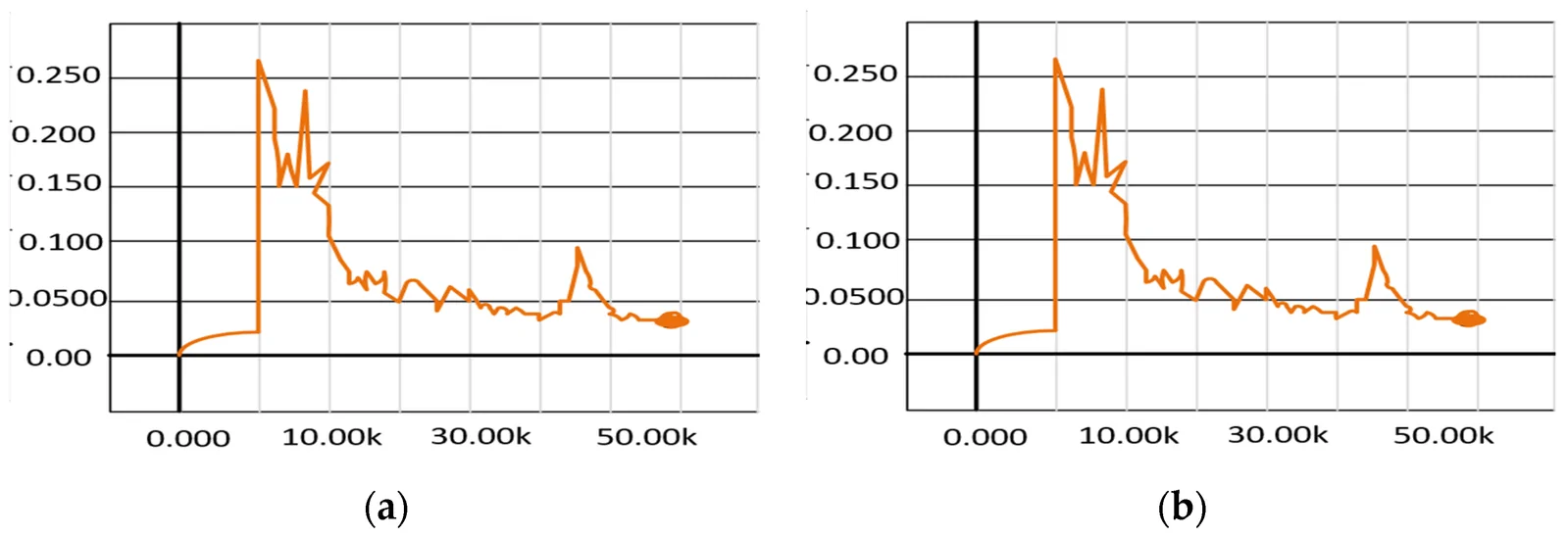
(**a**) The iteration during one hour of training (**b**) The training after completion of 1 h.

At the peak of 3.0, the Faster-RCNN-Inception-V2 model swiftly fell to a low of 0.8. It will take roughly 50,000 steps or three hours for the model to learn to lose less than 0.05 regularly. If a different model like the SSD-mobilenet-V1 model or rfcn_resnet101 is used for training, the loss numbers will be different as these models enable faster detection but at the expense of accuracy. A tensor board, which will open in a web browser, can be used to track the progress of the training job. Information on the training process can be found on the tensor design systems. Keeping track of the classifier’s overall loss over time is crucial. Every five minutes or so, the training routine saves checkpoints. If the training takes a more prolonged period of time, Ctrl + C is used in the command prompt window to stop it. Training can be paused and resumed anytime; it will resume from the last saved checkpoint. The frozen inference graph will be generated using the checkpoint with the most steps. Here, in this case, the inference graphs in Figure 14a,b depict the training after completion of 1 h. The overall loss over time is at the minimal stage, as the inference graph does not get linear. Following the completion of training, the frozen graph is formed. As the training process goes further, the iteration in the graph shows a change in loss as the graph starts getting linear.

Figure 14a illustrates the state of the model after one hour of training. The graph demonstrates how the loss function and accuracy metrics are evolving as the training progresses. Noticeable trends or patterns can provide insights into the model’s learning rate and potential areas of improvement. Figure 14b shows the training results upon the completion of one hour. This final state after the given duration highlights the overall progress made and indicates whether the model has converged, is still learning, or might be experiencing overfitting or underfitting.

**Figure 15:**
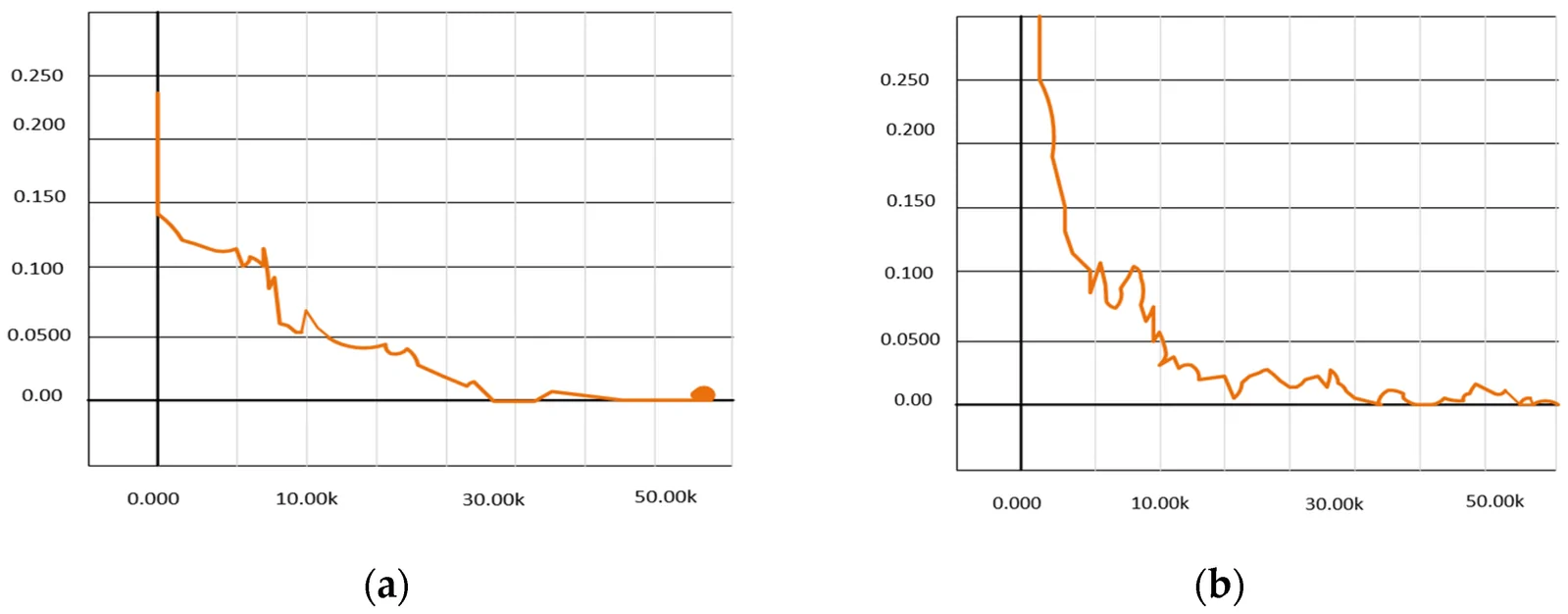
(**a**) Iteration during three hours of training (**b**) Iteration after completion of three hours of training.

Figure 15a illustrates the state of the model at an intermediate point after three hours of training. The graph displays the progression of the loss function and accuracy metrics over time, showing more advanced learning compared to Figure 14. Figure 15b presents the final state of the training after three hours, indicating the model’s performance upon reaching this extended duration. This part highlights the cumulative effect of the prolonged training period on the model’s learning and performance. Here, in this case, the inference graphs in Figure 15a,b depict the classifier’s overall loss over time. Following the completion of training, the frozen graph is formed. When the loss falls below 0.8, the line in the graph flattens, implying that the loss is minimal and the matching progress is high. Now, the real-time test can be carried out using random images, video, and a webcam. Replace the image name variable in Object detectionimage.py with the file name of the image you want to test to copy a photo of the object or objects into the object detection folder and test it.

### 4.1. Sinch Application for Sending Notification

**Figure 16:**
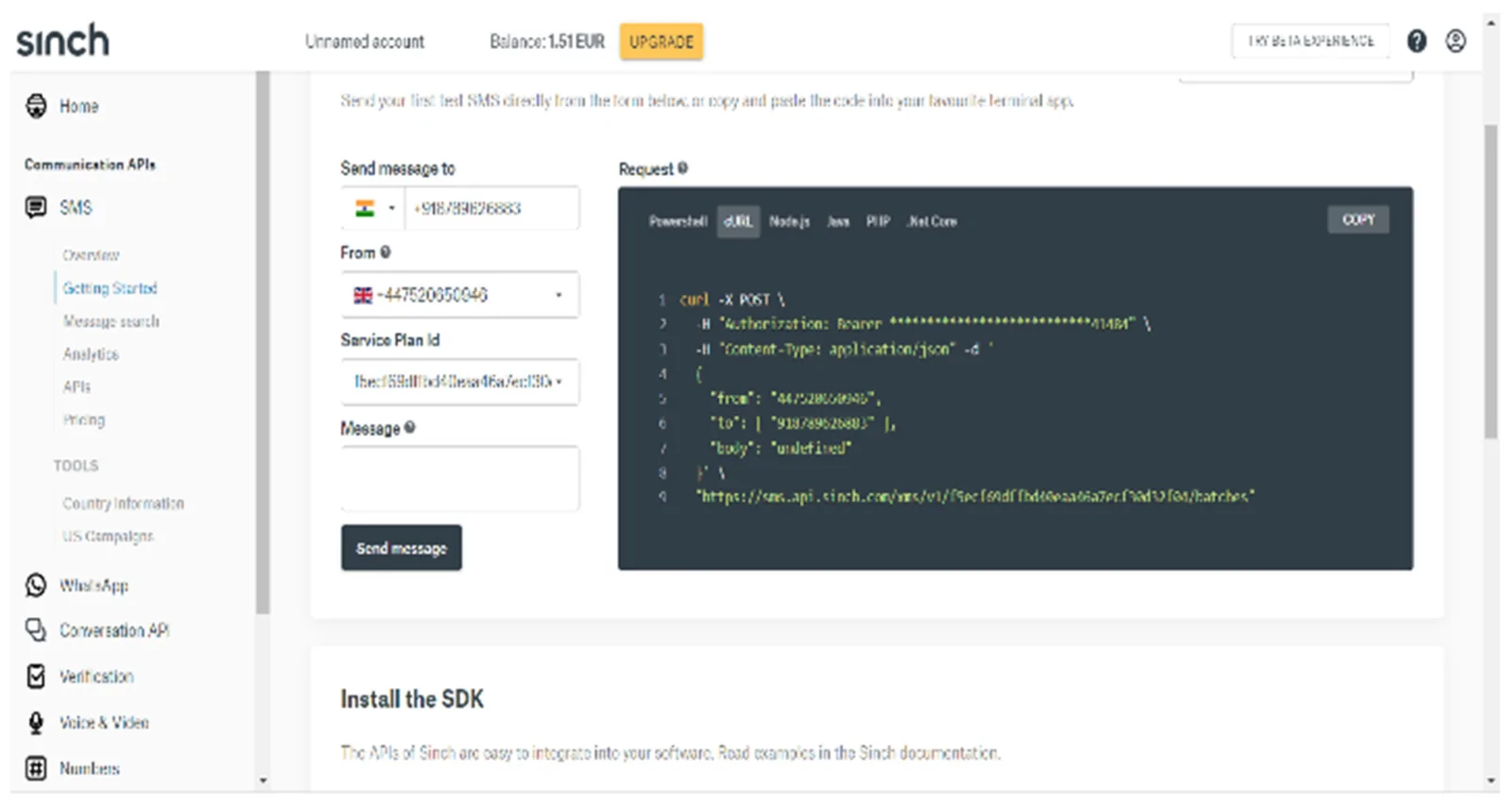
Sinch application for online Monitoring.

Sinch application is used for notification sending. This paper discusses creating a real-time image processing system with SMS notifications. Sinch is an SMS service provider, and their SMS service works similarly to the service of other SMS service providers. They connect their server computer to other SMS service providers and mobile telephone network operators using SMS protocols such as SMPP client connections, UCP/EMI protocol connections, and HTTP SMS or REST SMS connections. Sinch creates income by selling the SMS at a premium rate. Whenever the camera detects any face, the Sinch application sends a message to the user. The user must create a Sinch account before receiving the’ service plan ID’ and ‘token’ from them. Figure 16 shows the user ID created for sending notifications, and Figure 15b shows the Python program used for the function. After that, the user can enter the code. It is also necessary to change the sender and recipient numbers.

Employees can be protected from head injuries caused by objects, punctures, and extrusions by wearing PPE. According to a survey by the Bureau of Labor Statistics, more than eight in ten employees who sustained head injuries were not wearing headgear [3]. The study’s results revealed more than 90% accuracy when using CNN. This could be due to using a more advanced Tensorflow GPU v1.5 model and sufficient data for training. An effective early warning system through a notification can be implemented utilizing the provided method for real-time monitoring with high precision and recall. In addition, a UAV’s performance can be used as a leading indicator of overall site safety through active monitoring. In this paper, the development of UAVs, object detection technologies, and the methods for helmet detection are discussed. Previous research used specialized strategies to handle this problem, but they had limits in terms of adaptability and the construction site’s practical feasibility. Furthermore, given the limitations of practical use results, the capacity to identify workers who are not facing the cameras can be significantly improved by using deep learning. We studied the visual features of construction sites and analyzed the aspects affecting helmet detection while determining the best strategy for construction site applications. For its solid performance in object detection, the Faster R-CNN algorithm was chosen. To address earlier approach limitations, we tested Faster R-CNN’s performance on various construction site images. The test dataset included 500 images, and manual calibration was used to verify the precision and recall rates. The test dataset includes visual conditions such as viewing range, individual posture, and illumination on construction sites, which may be an issue. The results show that Faster R-CNN is robust in various visual circumstances on construction sites. Faster R-CNN, which uses a camera mounted on a drone to process images in real-time, has the potential to catch people whose heads are only partially visible when they move. Workers’ pixel-sized images, huge backgrounds, and many worker postures stand out in image frames taken from real-world far-field surveillance recordings on construction sites. Experiments have shown that faster R-CNN can recognize nhus from far-field images. At last, a simple technique is used for an alert system to recognize workers without a helmet, which is built in Python language using the Sinch application, and notifications are provided to the user after detection.

### 4.2. Summary of the Performance

For the effectiveness of the trained models, a confusion matrix was used. This matrix offers a thorough assessment of the models’ performance by comparing the interpretations made by humans with the predictions made by the models. The matrix included the number of true positives (TP), true negatives (TN), false positives (FP), and false negatives (FN) for each class. The evaluation of the Faster R-CNN (FRCNN) model for helmet detection yielded the following results: 3478 instances of true positives, indicating proper identification of helmets; 254 instances of false positives, indicating wrong detection of helmets; and 381 instances of false negatives, indicating the presence of helmets that were not identified. The model had a precision of 93.1%, which signifies the level of accuracy in its positive predictions. Additionally, the recall of the model was 90%, demonstrating its capability to identify all relevant cases. The measurements highlight the model’s excellent ability in detecting helmets, with high accuracy and recall rates. The use of tensorflow for image analysis in construction safety monitoring has shown considerable promise in improving adherence to PPE standards. Utilizing deep learning methodologies, namely CNN, the system proficiently categorizes and identifies critical safety items, including helmets, safety jackets, and other protective gear. The versatility of tensorflow enables the incorporation of several models customized to the distinct needs of monitoring different safety equipment in varied environmental circumstances. It is essential to acknowledge the limits of the existing methodology, especially regarding the resilience of the algorithms under diverse lighting conditions, occlusions, and site configurations. Given the unanticipated problems at construction sites, a proactive strategy that includes regular updates to the training dataset is crucial for ensuring the system’s correctness and durability. The developed system utilizing R-CNN achieved a precision of 93.1%, an mAP of 58.45%, and a frame rate of 27 FPS. Table 3 shows the comparison of the proposed work with the literature. 

Notwithstanding the gains, the research is constrained by the lack of hyperparameter optimization data, which impedes repeatability and performance assessment. The system’s emphasis on helmet detection may neglect other essential PPE elements, like gloves, footwear, and safety glasses, which are as vital for worker safety. The absence of comprehensive datasets including these factors further limits the system’s capacity to check compliance effectively. Moreover, the use of UAVs integrated with image recognition technologies may encounter regulatory obstacles and technical constraints in practical implementations.

## 5. Conclusions

Construction remains one of the world’s most dangerous professions to work in. Accidents cannot be predicted in the construction field and can occur at any place, time, and anywhere. The lack of knowledge among construction workers is a significant factor. Improved worker helmet detection is critical for properly managing on-site safety. For detecting helmets in far-field surveillance footage, this research recommends employing Faster R-CNN instead of the current approach currently in use. An enormous number of images were utilized for training a model called Faster RCNN to detect headwear as an initial step. After determining the visual characteristics of construction sites, the dataset was divided into two groups. It was then evaluated whether or not the proposed technique would work as intended in various site conditions. The RCNN-Inception model is faster, but it has lower detection accuracy. Due to the lower accuracy of these models, they are preferred over other algorithms in this case because they allow faster detection. This technology can enhance construction site monitoring and worker safety management in real-time. In addition, our technology can detect worker helmets but not the workers themselves. To respond to events with appropriate disciplinary measures and targeted safety training, research should concentrate on discovering and integrating data from workers into real-time monitoring systems for workplace safety. The developed system utilizing R-CNN achieved a precision of 93.1%, an mAP of 58.45%, and a frame rate of 27 FPS.

Future research should focus on enhancing the existing system’s capabilities to include the detection of other PPE components, including gloves, safety shoes, and goggles, so offering a more comprehensive perspective on worker safety compliance. This may be accomplished by integrating supplementary training data and enhancing the models to recognize different categories of safety equipment. Moreover, augmenting the algorithm’s flexibility to varying environmental circumstances and using sophisticated data augmentation methods might enhance its resilience. Furthermore, investigating real-time data analysis and incorporating feedback mechanisms might enhance the responsiveness of safety monitoring systems, hence fostering a safer working environment on construction sites.

## Figures and Tables

**Table 1:** Definitions True Positive, False Positive and False Negative for helmet detection.

Category	Real Helmet Detection	Predicted That Detection
FP	No	Yes
TP	Yes	Yes
FN	Yes	No

**Table 2:** Image sample information in various conditions.

Categories	No	Value	Total No of Workers Not Wearing Helmet	Number of Images
The Weather Situation				
	1	Rainy	75	500
	2	Cloudy	150	500
	3	Sunny	210	500
	4	Haze	275	500
Occlusions				
	1	Head visible	480	500
	2	Upper body visible	300	500
	3	Only part of head visible	128	500
	4	Whole body visible	198	500
Individual Posture				
	1	Sitting	75	500
	2	Standing	190	500
	3	Bending	175	500
	4	Squatting	86	500

**Table 3:** Comparison of Related Literature works.

Criteria	Drone-Assisted Camera Networks for 3D Terrain [39]	UAVs for Safety Inspection on Construction Sites [40]	UAVs for Civil Infrastructure Applications [41]	Proposed (UAV for Helmet Detection in Construction)
Application	Drone-assisted camera networks for terrain deployment	Safety inspection on construction sites	UAVs in civil infrastructure, including post-disaster and monitoring	Helmet detection for safety on construction sites
Objective	Optimize camera deployment for 3D environments using an improved evolutionary algorithm	Identify and inspect non-compliance with safety regulations	Summarize UAV applications and sensor payloads	Real-time helmet detection to enhance worker safety
Key Technology/Method	Many-objective optimization with improved evolutionary algorithm and Gaussian process regression	Protocol for UAS flights and data processing for visual assets	UAV types, sensor payloads, and wireless sensor networks	Tensorflow and Faster R-CNN for real-time helmet detection
Outcome	Improved deployment strategies for drone-assisted networks	Improved safety monitoring and compliance on jobsites	Overview of UAVs in infrastructure and recommendations	Increased accuracy, recall, and cost-effectiveness for safety compliance
Innovative Feature	Use of Gaussian process regression and quantized mutation operator in optimization	Development of guidelines for UAV-based safety inspection	Detailed guidance for researchers and emerging trends	Automated safety compliance system for construction workers

## Data Availability

All the data used for the analysis are available within the manuscript.
