# Correction: Kumar et al. Investigation of Unsafe Construction Site Conditions Using Deep Learning Algorithms Using Unmanned Aerial Vehicles. *Sensors* 2024, *24*, 6737

**DOI:** 10.3390/s26154668

**Published:** 2026-07-23

**Authors:** Sourav Kumar, Mukilan Poyyamozhi, Balasubramanian Murugesan, Narayanamoorthi Rajamanickam, Roobaea Alroobaea, Waleed Nureldeen

**Affiliations:** 1 Department of Civil Engineering, SRM Institute of Science and Technology, Kattankulathur, Chennai 603203, India; sourav.kumar390@gmail.com (S.K.); mp6481@srmist.edu.in (M.P.); 2 Department of Electrical and Electronics Engineering, SRM Institute of Science and Technology, Kattankulathur, Chennai 603203, India; narayanr@srmist.edu.in; 3 Department of Computer Science, College of Computers and Information Technology, Taif University, P.O. Box 11099, Taif 21944, Saudi Arabia; r.robai@tu.edu.sa; 4 General Subject Department, University of Business and Technology, Jeddah 23435, Saudi Arabia

## 1. References Correction

In the original publication [1], References 1, 2, 4, 6, 8, 10–12, 14, 16–20, 22, 24–26, 28, 30, 32–34, 36–38, 40, 44–56 were identified as not directly supporting the corresponding in-text statements and have therefore been revised or removed where appropriate. references have been added that better support the in-text claims, listed as References 2, 3, 5–10, 12, 14, 15, 18, 20, 23, 24, 29, 30, 33, 35, 39, 41 in the revised manuscript. The following references from the original version have been renumbered: 3 to 1, 5 to 4, 7 to 22, 9 to 13, 13 to 17, 15 to 40, 21 to 38, 23 to 19, 27 to 21, 29 to 26, 31 to 34, 35 to 11, 39 to 25, 41 to 28, 42 to 16, 43 to 27, 57 to 31, 58 to 37, 59 to 36, and 60 to 32.

2. Newaz, M.T.; Ershadi, M.; Jefferies, M.; Davis, P. A critical review of the feasibility of emerging technologies for improving safety behavior on construction sites. *J. Saf. Res.* **2024**, *89*, 269–287. https://doi.org/10.1016/j.jsr.2024.04.006.

3. Occupational Safety and Health Administration (OSHA). Personal Protective Equipment. OSHA Publication 3151-12R. U.S. Department of Labor, Washington, DC, 2003. Available online: https://www.osha.gov/sites/default/files/publications/osha3151.pdf (accessed on 10 October 2024)

5. Onososen, A.O.; Musonda, I.; Onatayo, D.; Tjebane, M.M.; Saka, A.B.; Fagbenro, R.K. Impediments to Construction Site Digitalisation Using Unmanned Aerial Vehicles (UAVs). *Drones* **2023**, *7*, 45. https://doi.org/10.3390/drones7010045.

6. Wang, X.; Yang, Y.; Chan, A.P.; Chi, H.-L.; Yung, E.H. A regulatory framework for the use of small unmanned aircrafts (SUAs) in the construction industry. *Eng. Constr. Arch. Manag.* **2023**, *31*, 3024–3049. https://doi.org/10.1108/ecam-10-2022-0990.

7. Krook, J.; Bossens, D.; Winter, P.; Araujo-Estrada, S.; Downer, J.; Windsor, S. Mapping the Complexity of Legal Challenges for Trustworthy Drones on Construction Sites in the United Kingdom. *ACM J. Responsible Comput.* **2024**, *1*, 1–26. https://doi.org/10.1145/3664617.

8. Rakha, T.; Gorodetsky, A. Review of Unmanned Aerial System (UAS) applications in the built environment: Towards automated building inspection procedures using drones. *Autom. Constr.* **2018**, *93*, 252–264. https://doi.org/10.1016/j.autcon.2018.05.002.

9. Zhen, L.; Yang, Z.; Laporte, G.; Yi, W.; Fan, T. Unmanned Aerial Vehicle Inspection Routing and Scheduling for Engineering Management. *Engineering* **2024**, *36*, 223–239. https://doi.org/10.1016/j.eng.2023.10.014.

10. Rehman, M.S.U.; Shafiq, M.T.; Ullah, F. Automated Computer Vision-Based Construction Progress Monitoring: A Systematic Review. *Buildings* **2022**, *12*, 1037. https://doi.org/10.3390/buildings12071037.

12. Xiao, C.; Wang, B.; Zhao, D.; Wang, C. Comprehensive investigation on Lithium batteries for electric and hybrid-electric unmanned aerial vehicle applications. *Therm. Sci. Eng. Prog.* **2023**, *38*, 101677. https://doi.org/10.1016/j.tsep.2023.101677.

14. Gupta, S.; Nair, S. A review of the emerging role of UAVs in construction site safety monitoring. *Mater. Today Proc.* **2024**. https://doi.org/10.1016/j.matpr.2023.03.135.

15. Liang, C.-J.; Le, T.-H.; Ham, Y.; Mantha, B.R.; Cheng, M.H.; Lin, J.J. Ethics of artificial intelligence and robotics in the architecture, engineering, and construction industry. *Autom. Constr.* **2024**, *162*, 105369. https://doi.org/10.1016/j.autcon.2024.105369.

18. Peksa, J.; Mamchur, D. A Review on the State of the Art in Copter Drones and Flight Control Systems. *Sensors* **2024**, *24*, 3349. https://doi.org/10.3390/s24113349.

20. Guo, B.H.; Zou, Y.; Fang, Y.; Goh, Y.M.; Zou, P.X. Computer vision technologies for safety science and management in construction: A critical review and future research directions. *Saf. Sci.* **2021**, *135*, 105130. https://doi.org/10.1016/j.ssci.2020.105130.

23. Elmeseiry, N.; Alshaer, N.; Ismail, T. A Detailed Survey and Future Directions of Unmanned Aerial Vehicles (UAVs) with Potential Applications. *Aerospace* **2021**, *8*, 363. https://doi.org/10.3390/aerospace8120363.

24. Samsami, R. A Systematic Review of Automated Construction Inspection and Progress Monitoring (ACIPM): Applications, Challenges, and Future Directions. *CivilEng* **2024**, *5*, 265–287. https://doi.org/10.3390/civileng5010014.

29. Dalal, N.; Triggs, B. Histograms of Oriented Gradients for Human Detection. In Proceedings of the Computer Vision and Pattern Recognition, San Diego, CA, USA, 20–26 June 2005; pp. 886–893. https://doi.org/10.1109/CVPR.2005.177.

30. Felzenszwalb, P.F.; Girshick, R.B.; McAllester, D.; Ramanan, D. Object Detection with Discriminatively Trained Part-Based Models. *IEEE Trans. Pattern Anal. Mach. Intell.* **2009**, *32*, 1627–1645. https://doi.org/10.1109/tpami.2009.167.

33. Rianto, Y. Road network detection from SPOT satellite image using Hough transform and optimal search. In Proceedings of the Asia-Pacific Conference on Circuits and Systems, Denpasar, Indonesia, 28–31 October 2002; pp. 177–180. https://doi.org/10.1109/APCCAS.2002.1115148.

35. Ren, S.; He, K.; Girshick, R.; Sun, J. Faster R-CNN: Towards real-time object detection with region proposal networks. *IEEE Trans. Pattern Anal. Mach. Intell.* **2017**, *39*, 1137–1149. https://doi.org/10.1109/TPAMI.2016.2577031.

39. Nex, F.; Remondino, F. UAV for 3D mapping applications: A review. *Appl. Geomat.* **2014**, *6*, 1–15. https://doi.org/10.1007/s12518-013-0120-x.

41. Outay, F.; Mengash, H.A.; Adnan, M. Applications of unmanned aerial vehicle (UAV) in road safety, traffic and highway infrastructure management: Recent advances and challenges. *Transp. Res. Part A Policy Pract.* **2020**, *141*, 116–129. https://doi.org/10.1016/j.tra.2020.09.018.

With this correction, the order of some references has been adjusted accordingly.

## 2. Text Correction

1. In the first Paragraph of Section 1. The content “To mitigate these ongoing safety problems, innovative technology such as UAVs is being incorporated into construction safety processes.” Has been updated to “To mitigate these ongoing safety problems, innovative technology such as UAVs is being incorporated into construction safety processes [1].”
2. In the 1st Paragraph of Section 1. The content “This research seeks to examine the obstacles associated with the application and potential of UAVs in enhancing safety on building sites, while also resolving the inherent difficulties [1]” has been updated to “This research seeks to examine the obstacles associated with the application and potential of UAVs in enhancing safety on building sites, while also resolving the inherent difficulties.”
3. In the 1st Paragraph of Section 1. The content “Notwithstanding extensive attempts to enhance worker safety, data reveal that construction remains responsible for a considerable proportion of occupational injuries and deaths globally. According to the U.S. Bureau of Labor Statistics (BLS), 84% of employees who had impact injuries were not using appropriate PPE.” has been updated to “Notwithstanding extensive attempts to enhance worker safety, data reveal that construction remains responsible for a considerable proportion of occupational injuries and deaths globally [2]. According to the U.S. Bureau of Labor Statistics (BLS), 84% of employees who had impact injuries were not using appropriate PPE [3].”
4. In the 1st Paragraph of Section 1. The content “In this environment, conventional techniques of monitoring safety compliance, such as site supervisors doing physical inspections, have shown inadequacy in both effectiveness and efficiency.” has been updated to “In this environment, conventional techniques of monitoring safety compliance, such as site supervisors doing physical inspections, have shown inadequacy in both effectiveness and efficiency [4].”
5. In the 1st Paragraph of Section 1. The content “These rules sometimes set limitations on the locations, timings, and methods of drone operation, potentially conflicting with the requirements of a building site that needs ongoing surveillance.” has been updated to “These rules sometimes set limitations on the locations, timings, and methods of drone operation, potentially conflicting with the requirements of a building site that needs ongoing surveillance [6].”
6. In the 1st Paragraph of Section 1. The content “Despite the user-friendly design of many contemporary UAVs, proficient operation requires a specific skill set, especially when managing bigger, more intricate drones equipped with sophisticated functionalities like thermal imaging, 3D mapping, and autonomous flying capabilities.” has been updated to “Despite the user-friendly design of many contemporary UAVs, proficient operation requires a specific skill set, especially when managing bigger, more intricate drones equipped with sophisticated functionalities like thermal imaging, 3D mapping, and autonomous flying capabilities [8].”
7. In the 1st Paragraph of Section 1. The content “The problem lies in ensuring that UAV operators possess not just flying proficiency but also training in construction safety regulations to accurately evaluate and respond to the gathered data [6,7].” has been updated to “The problem lies in ensuring that UAV operators possess not just flying proficiency but also training in construction safety regulations to accurately evaluate and respond to the gathered data.”
8. In the 1st Paragraph of Section 1. The content “Collecting this data are only the first step; it must then be processed, evaluated, and acted upon in real time.” has been updated to “Collecting this data are only the first step; it must then be processed, evaluated, and acted upon in real time [10].”
9. In the 1st Paragraph of Section 1. The content “Establishing the requisite data processing systems may be both labor-intensive and costly, especially for smaller enterprises or projects with constrained budgets [10]. Moreover, UAVs are vulnerable to environmental factors, especially meteorological conditions.” has been updated to “Establishing the requisite data processing systems may be both labor-intensive and costly, especially for smaller enterprises or projects with constrained budgets. Moreover, UAVs are vulnerable to environmental factors, especially meteorological conditions [11].”
10. In the 1st Paragraph of Section 1. The content “Construction has historically been a labor-intensive sector, and several workers and supervisors may be reluctant to adopt technology that may be seen as jeopardizing job security or introducing unwarranted complexity to existing processes.” Has been updated to “Construction has historically been a labor-intensive sector, and several workers and supervisors may be reluctant to adopt technology that may be seen as jeopardizing job security or introducing unwarranted complexity to existing processes [13].”
11. In the 1st Paragraph of Section 1. The content “UAVs outfitted with high-resolution cameras may unintentionally record images of adjacent private properties or persons, hence presenting concerns around data protection and privacy infringements.” has been updated to “UAVs outfitted with high-resolution cameras may unintentionally record images of adjacent private properties or persons, hence presenting concerns around data protection and privacy infringements [15].”
12. In the 1st Paragraph of Section 1. The content “Construction firms must guarantee that their use of UAVs adheres to all relevant privacy regulations, and they may need to formulate explicit protocols for drone operation to prevent legal issues [15,16].” has been updated to “Construction firms must guarantee that their use of UAVs adheres to all relevant privacy regulations, and they may need to formulate explicit protocols for drone operation to prevent legal issues.”
13. In the 1st Paragraph of Section 1. The content “UAVs are fundamentally airborne computing systems, and like any digital apparatus, they are vulnerable to hacking or illegal intrusion.” has been updated to “UAVs are fundamentally airborne computing systems, and like any digital apparatus, they are vulnerable to hacking or illegal intrusion [16].”
14. In the 1st Paragraph of Section 1. The content “As the industry advances towards further automation via robotics, Building Information Modeling (BIM), and the Internet of Things (IOT), UAVs are expected to become a fundamental component of the construction ecosystem. Their capacity to provide real-time data and insights will enhance other digital technologies, contributing to the development of safer, more efficient, and more sustainable building projects [23].” has been updated to “As the industry advances towards further automation via robotics, Building Information Modeling (BIM), and the Internet of Things (IOT), UAVs are expected to become a fundamental component of the construction ecosystem [22]. Their capacity to provide real-time data and insights will enhance other digital technologies, contributing to the development of safer, more efficient, and more sustainable building projects.”
15. In the 1st Paragraph of Section 1. The content “A primary challenge is maneuvering through intricate legal frameworks that impose limitations on UAV operations in urban and high-risk zones [28,29].” has been updated to “A primary challenge is maneuvering through intricate legal frameworks that impose limitations on UAV operations in urban and high-risk zones.”
16. In the 1st Paragraph of Section 1. The content “Technical constraints, like limited battery longevity, susceptibility to weather conditions, and data processing demands, exacerbate the challenges of implementation [30,31].” has been updated to “Technical constraints, like limited battery longevity, susceptibility to weather conditions, and data processing demands, exacerbate the challenges of implementation.”
17. In the 1st Paragraph of Section 1. The content “Ethical considerations, including privacy and cybersecurity threats, must be addressed to guarantee appropriate UAV utilization [32].” has been updated to “Ethical considerations, including privacy and cybersecurity threats, must be addressed to guarantee appropriate UAV utilization.”
18. In the 1st Paragraph of Section 2. The content “These models function by identifying pertinent properties related to helmets, such as form, color, and texture, facilitating precise recognition even under the dynamic conditions typical of construction sites.” has been updated to “These models function by identifying pertinent properties related to helmets, such as form, color, and texture, facilitating precise recognition even under the dynamic conditions typical of construction sites [25].”
19. In the 1st Paragraph of Section 2. The content “Fluctuations in these factors may result in varying results in helmet detection accuracy, thereby compromising the dependability of the monitoring system [33,34].” has been updated to “Fluctuations in these factors may result in varying results in helmet detection accuracy, thereby compromising the dependability of the monitoring system.”
20. In the 1st Paragraph of Section 2. The content “Recognizing and mitigating this restriction, while ensuring appropriate processing time, is crucial for evaluating the efficacy and dependability of this novel safety management strategy [39,40].” has been updated to “Recognizing and mitigating this restriction, while ensuring appropriate processing time, is crucial for evaluating the efficacy and dependability of this novel safety management strategy.”
21. In the 1st Paragraph of Section 2.1. The content “The study utilizes the F450 Quadcopter UAV, a robust and versatile unmanned aerial vehicle equipped with an object tracking camera to monitor construction sites [41]. The UAV’s aerial capabilities provide a comprehensive view of the site, enabling it to navigate complex environments and capture high-resolution images and video footage [42,43].” has been updated to “The study utilizes the F450 Quadcopter UAV, a robust and versatile unmanned aerial vehicle equipped with an object tracking camera to monitor construction sites. The UAV’s aerial capabilities provide a comprehensive view of the site, enabling it to navigate complex environments and capture high-resolution images and video footage.”
22. In the 1st Paragraph of Section 2.1. The content “For this study, images of workers with and without helmets are collected from real-time construction sites [44,45].” has been updated to “For this study, images of workers with and without helmets are collected from real-time construction sites.”
23. In the 1st Paragraph of Section 2.3. The content “As the UAV patrols the construction site, the camera captures real-time video footage, which is fed into the tensorflow model [47,48].” has been updated to “As the UAV patrols the construction site, the camera captures real-time video footage, which is fed into the tensorflow model.”
24. In the 2nd Paragraph of Section 2.3. The content “The main objective of the UAV is to assess if workers are wearing helmets, which are an essential component of PPE required to mitigate head accidents at construction sites [49].” has been updated to “The main objective of the UAV is to assess if workers are wearing helmets, which are an essential component of PPE required to mitigate head accidents at construction sites.”
25. In the 1st Paragraph of Section 2.4. The content “The Sky Stars F405 Flight Controller has a dedicated 10V output for the DJI air unit and a typical analog video on-screen display (OSD). This board also includes a 640 × 480 optic flow camera [6].” has been updated to “The Sky Stars F405 Flight Controller has a dedicated 10V output for the DJI air unit and a typical analog video on-screen display (OSD). This board also includes a 640 × 480 optic flow camera.”
26. In the 1st Paragraph of Section 2.4. The content “That connects various interfaces for electronic speed controllers (ESC), remote controls, and GPS module connectors that output a micro-air vehicle communication protocol to ensure compatibility with all fixed-wing and rotor UAV frames while conserving IO ports (mavlink) [50].” has been updated to “That connects various interfaces for electronic speed controllers (ESC), remote controls, and GPS module connectors that output a micro-air vehicle communication protocol to ensure compatibility with all fixed-wing and rotor UAV frames while conserving IO ports (mavlink).”
27. In the 1st Paragraph of Section 2.4. The content “Visual object recognition is ineffective if an image lacks the desired elements [51,52]. HOG (Histograms of oriented gradients) was previously used for pedestrian identification by Dalal et al. In 2005.” Has been updated to “Visual object recognition is ineffective if an image lacks the desired elements. HOG (Histograms of oriented gradients) was previously used for pedestrian identification by Dalal et al. In 2005 [29].”
28. In the 1st Paragraph of Section 2.5. The content “Among these models, Faster R-CNN (Region-Based Convolutional Neural Network) stands out due to its efficiency in real-time object recognition.” has been updated to “Among these models, Faster R-CNN (Region-Based Convolutional Neural Network) stands out due to its efficiency in real-time object recognition [31].”
29. In the 2nd Paragraph of Section 2.7. The content “This feature extraction utilizes sophisticated machine learning models, perhaps using frameworks like tensorflow and deep learning architectures such as Convolutional Neural Networks (CNNs), Faster R-CNN, or R-CNN.” has been updated to “This feature extraction utilizes sophisticated machine learning models, perhaps using frameworks like tensorflow and deep learning architectures such as Convolutional Neural Networks (CNNs), Faster R-CNN, or R-CNN [32].”
30. In the 2nd Paragraph of Section 2.7. The content “The approach may also include the Hough Transform algorithm, which is proficient at detecting geometric patterns, particularly the circular shape typical of helmets.” has been updated to “The approach may also include the Hough Transform algorithm, which is proficient at detecting geometric patterns, particularly the circular shape typical of helmets [33].”
31. In the 1st Paragraph of Section 3. The content “In image analysis, tensorflow is especially adept at training CNN to identify and categorize items, such as safety helmets, coats, and cars, which is essential for maintaining compliance with safety rules in hazardous work conditions.” has been updated to “In image analysis, tensorflow is especially adept at training CNN to identify and categorize items, such as safety helmets, coats, and cars, which is essential for maintaining compliance with safety rules in hazardous work conditions [34].”
32. In the 1st Paragraph of Section 3. The content “The training procedure entails inputting the labeled information into the CNN, enabling the model to discern patterns and characteristics linked to each class [54,55].” has been updated to “The training procedure entails inputting the labeled information into the CNN, enabling the model to discern patterns and characteristics linked to each class.”
33. In the 1st Paragraph of Section 3.1. The content “A box is created around each object in each image in the directory. Labeling 7 makes an an.xml file for testing and training [56,57].” has been updated to “A box is created around each object in each image in the directory. Labeling 7 makes an an.xml file for testing and training.”
34. In the 1st Paragraph of Section 3.2. The content “Once trained, the model is used as a basis for the final algorithm (Faster RCNN), and analysis [58] is followed by labeling of images, formation of bounding boxes in images, and preparation of data explained below. The Faster R-CNN object identification algorithm was introduced by Ren et al. in 2015.” has been updated to “Once trained, the model is used as a basis for the final algorithm (Faster RCNN), and analysis is followed by labeling of images, formation of bounding boxes in images, and preparation of data explained below. The Faster R-CNN object identification algorithm was introduced by Ren et al. in 2015 [35].”
35. In the 2nd Paragraph of Section 3.2. The content “The efficacy of Faster R-CNN in identifying helmet use among workers enables real-time analysis of video feeds from UAVs, delivering prompt feedback to verify adherence to safety rules.” has been updated to “The efficacy of Faster R-CNN in identifying helmet use among workers enables real-time analysis of video feeds from UAVs, delivering prompt feedback to verify adherence to safety rules [37].”
36. In the 2nd Paragraph of Section 4. The content “The Faster RCNN model was tested on a wide variety of images. Following is a breakdown of the accuracy in various conditions [60].” has been updated to “The Faster RCNN model was tested on a wide variety of images. Following is a breakdown of the accuracy in various conditions.”
37. In the 5th Paragraph of Section 5. The content “Under several extractions, the original image extracts. R-CNN architecture, developed by Girshick and colleagues, produces 500 bounding boxes from a photo and uses a CNN technique to extract the bounding box features.” has been updated to “Under several extractions, the original image extracts. R-CNN architecture, developed by Girshick and colleagues, produces 500 bounding boxes from a photo and uses a CNN technique to extract the bounding box features [38].”
38. In the 2nd Paragraph of Section 4.1. The content “According to a survey by the Bureau of Labor Statistics, more than eight in ten employees who sustained head injuries were not wearing headgear.” has been updated to “According to a survey by the Bureau of Labor Statistics, more than eight in ten employees who sustained head injuries were not wearing headgear [3].”
39. In the 2nd Paragraph of Section 4.1. The content “At last, a simple technique is used for an alert system to recognize workers without a helmet [39], which is built in Python language using the Sinch application, and notifications are provided to the user after detection.” has been updated to “At last, a simple technique is used for an alert system to recognize workers without a helmet, which is built in Python language using the Sinch application, and notifications are provided to the user after detection.”

The authors state that the scientific conclusions are unaffected. This correction was approved by the Academic Editor. The original publication has also been updated.

## References

[1] Kumar S., Poyyamozhi M., Murugesan B., Rajamanickam N., Alroobaea R., Nureldeen W. (2024). Investigation of Unsafe Construction Site Conditions Using Deep Learning Algorithms Using Unmanned Aerial Vehicles. Sensors.

